# Assessment of Pesticide Residue Content in Fresh Plant-Based Products Available on the Serbian Market Using the QuEChERS Method Combined with LC-MS/MS and GC-MS/MS

**DOI:** 10.3390/foods15122081

**Published:** 2026-06-08

**Authors:** Danica Mrkajić, Isidora Kecojević, Vladimir Tomović, Biljana Bajić, Milana Lazović, Ana Joksimović, Aleksandra Martinović, Dragan Vujadinović, Milena Terzić, Vesna Đorđević

**Affiliations:** 1 Faculty of Technology Novi Sad, University of Novi Sad, 21000 Novi Sad, Serbia; danica.mrkajic@abiotechlab.rs (D.M.); isidora.kecojevic@abiotechlab.rs (I.K.); milana.lazovic@abiotechlab.rs (M.L.); milenavujanovic@uns.ac.rs (M.T.); 2 A Bio Tech Lab d.o.o., 21208 Sremska Kamenica, Serbia; biljana.bajic@abiotechlab.rs (B.B.); ana.joksimovic@abiotechlab.rs (A.J.); 3 Faculty for Food Technology, Food Safety and Ecology, University of Donja Gorica, Donja Gorica, 81000 Podgorica, Montenegro; aleksandra.martinovic@udg.edu.me; 4 Faculty of Technology Zvornik, University of East Sarajevo, 75400 Zvornik, Bosnia and Herzegovina; dragan.vujadinovic@tfzv.ues.rs.ba; 5 Institute of Meat Hygiene and Technology, 11040 Belgrade, Serbia; vesna.djordjevic@inmes.rs

**Keywords:** vegetables, pesticide residues, maximum residue levels, food safety

## Abstract

Pesticides play a crucial role in modern agriculture by protecting crops from pests, diseases, and weeds, thereby contributing to increased agricultural productivity and food security. However, their extensive use may lead to the presence of residues in food products, particularly vegetables, which can pose potential risks to human health. Therefore, continuous monitoring of pesticide residues in vegetables is essential to ensure food safety, assess dietary exposure, and protect consumers from possible acute and chronic health effects associated with pesticide intake. In this study, the concentrations of pesticide residues were determined in 1236 samples of 44 vegetable species collected over a four-year period. Vegetables originated from 39 countries, including Serbia (*n* = 213). Pesticide residues were determined by liquid chromatography–tandem mass spectrometry (LC-MS/MS) and gas chromatography–tandem mass spectrometry (GC-MS/MS) after extraction using a modified QuEChERS protocol. A total of 148 pesticide residues were detected. Of the vegetable samples, 40.13% had pesticide residues at or above 0.01 mg/kg, and 1.78% exceeded the maximum residue limits (MRLs) set by the Serbian regulation. MRL values were most often exceeded in ginger, cucumber, and spinach. The most frequently found pesticide was imidacloprid (detected in 74 samples, 5.99%). Multiple pesticides were detected in 22.01% of the vegetable samples, and one tomato sample contained up to 10 pesticide residues. Based on the available data and further development of a representative dataset, together with appropriate statistical analyses, dietary exposure assessments for pesticides can be conducted.

## 1. Introduction

Owing to their many health benefits, vegetables represent one of the most important foundations in the human diet. People consume the roots, stalks, flowers, leaves and other sections of these annual and perennial horticultural crops in different ways—whether as a whole or only in part, either cooked or raw. The health benefits of vegetables stem from the high content of vitamins, minerals, dietary fibre and phytochemical compounds they contain. The presence of dietary fibres and antioxidant vitamins, such as vitamin A, vitamin C and vitamin E, is particularly beneficial for human health. Some of the health benefits of a vegetable-based diet include the prevention of many chronic illnesses, in particular cardiovascular diseases, obesity, diabetes, metabolic syndrome, and cancer, and lowering the risk factors associated with these illnesses [1]. The minimal amount of vegetables and fruits that lowers the risk of certain noncommunicable diseases as prescribed by the World Health Organization [2] is 400 g per day. In 2023, global vegetable and fruit production reached 2.1 billion tons, whereas in 1961 it amounted to nearly 400 million tons [3].

Pesticides are widely used in modern agriculture and food production mainly due to their ability to increase agricultural productivity and to protect crops from diseases and pests, namely insects, rodents, bacteria, weeds or fungi. Among the many benefits of using pesticides is their ability to increase the potential of the repeated growth of the same crop on the same soil during the year. However, being designed to interfere with biological processes, they can be harmful for humans depending on the degree and method of a person’s exposure, causing both acute and chronic health problems [4,5,6,7]. Both direct and indirect exposure to pesticides has the potential to cause severe toxicity in humans, leading to different chronic illnesses, diabetes, asthma, Parkinson’s disease, leukemia, cancer, cognitive impairment and suicidal tendencies. Different methods of exposure include occupational exposure of workers in the agricultural industry or the chemical industry producing pesticides, chemical accidents in the industry or even mass food poisonings from increased levels of pesticides in food. The use of pesticides is, for example, responsible for three million cases of poisonings, 220,000 deaths, and around 750,000 cases of chronic illnesses annually worldwide, with developing countries accounting for most of these cases [8,9]. Therefore, it is essential that the use of pesticides is regulated by national and European legal frameworks. This means that broad and detailed systems of laws and regulations are responsible for specifying different aspects of the use of pesticides, such as whether certain active substances can be approved, whether they can be used in plant protection products, and the permissible levels of their residues in food. Regulation (EC) No 396/2005 [10], for example, specifies the so-called ‘maximum residue levels’ (MRLs), which represent the legally permissible limits of pesticides, with the aim of achieving a high standard of protecting consumers from the toxic effects of these bioactive substances. More than 1300 pesticides and 378 food products/food groups [11] are included in the European Union harmonized MRLs. In order to address emerging safety risks, several national regulations and the European Commission regulations are in place to provide ongoing amendments of the law by including new food products/commodities/pesticides and updating the permissible levels based on current findings gathered by the authorities in the EU member states, by the European Food Safety Authority (EFSA), and by the European Commission. During the past 10 years, the law on MRLs of pesticides in food in Serbia has undergone several amendments [12,13,14] in order to conform to the EU legal standards. The most recent version of the Regulation [15] on the Maximum Residue Levels of Pesticides in Food in Serbia is entirely aligned with the European Union safety standards.

A number of studies investigating the level of pesticide residues in vegetables and food products have been conducted in recent years [16,17,18,19,20,21,22,23,24,25,26,27,28,29,30,31,32,33,34,35,36,37,38,39,40,41,42,43,44,45,46,47,48,49,50,51,52,53,54,55,56,57,58,59,60,61,62,63,64,65,66,67,68,69,70,71,72,73,74,75,76,77,78,79,80,81,82,83,84,85,86,87,88,89,90,91,92,93,94,95,96,97,98,99,100,101]. Supplementary Material S2, Table S1, gives a summary of the available literature data on pesticide residues in vegetables starting from the year 2010 onwards. These studies largely indicate that pesticide residues are currently very commonly detected in vegetables and food. Although the levels of pesticide residues are shown to frequently exceed the limit of quantification (LOQ), the values detected rarely exceed the MRL. Moreover, these studies focus on the highly toxic pesticides whose use is banned in many countries. All of this leads to the conclusion that in order to minimize or eliminate food safety risks for human health, adequate pesticide residue control measures must be implemented.

The analysis of pesticide residue levels usually consists of several stages, namely sample preparation (homogenization, extraction, and clean-up), separation, detection, and data analysis [7]. Among these different steps, the process of extraction is critically important since it largely affects the correct quantification of pesticide residues. The most common extraction methods are liquid–liquid extraction (LLE), solid-phase extraction (SPE), matrix solid-phase dispersion (MSPD), quick, easy, cheap, effective, rugged, and safe (QuEChERS) extraction, and solid-phase microextraction (SPME) [7]. The sample preparation technique based on the QuEChERS extraction is widely applied in the multiclass or multiresidue analysis of different types of pesticides mainly used in agriculture/foodstuff/vegetable/fruit [7,17,18,19,20,21,22,23,24,25,26,27,28,29,30,31,32,33,34,35,36,37,38,39,40,41,42,43,44,45,46,47,48,49,50,51,52,53,102]. Over the past two decades, the analysis of pesticide residues in food has attracted considerable attention among researchers, and a considerable number of detection methods have been proposed. The traditional analytical methods include gas chromatography (GC) and high-performance liquid chromatography (HPLC) coupled with various detectors [electron capture detector (GC-ECD), flame photometric detector (GC-FPD), nitrogen phosphorus detector (GC-NPD), and flame ionization detector (GC-FID); UV detection (HPLC-UV), fluorescence detector (HPLC-FD), and diode-array detector (HPLC-DAD)]. Apart from these, multi-pesticide residue analysis is very commonly based on liquid chromatography–mass spectrometry (LC-MS). It is possible to achieve high sensitivity and selectivity without derivatization through the MS (and tandem mass spectrometry—MS/MS) detection system. Identification and confirmation can be conducted in a single step when applied to LC [7,18,19,20,21,22,28,29,31,33,34,35,36,38,39,41,43,44,45,49,50,51,52,53,54,55,56,57,58,59,60,61,62,63,64,102].

This paper is part of research focused on the presence of pesticide residues in food/vegetables in the Republic of Serbia [16].

The objective of this study was to investigate the concentrations of pesticide residues in vegetables, which are collected as a part of the national monitoring programme for pesticide residues in Serbia, and to compare these levels with maximum residue levels established by the Serbian regulation [12,13].

## 2. Materials and Methods

### 2.1. Sampling

Over a 4-year period (2016–2019), concentrations of pesticide residues were determined in 1236 independent samples of fresh vegetables. These analyses were performed at the request of the Ministry of Agriculture, Forestry and Water of the Republic of Serbia in an accredited laboratory that has a contract with the government (A Bio Tech Lab) before the products were placed on the market.

Primary samples of each vegetable were taken from eight different packages, all of the same provenance, and six vegetables were taken randomly from each package. The following parts of the vegetable were used for homogenization: whole product after removal of root, decayed leave and soil (arugula, cornsalad, leek, lettuce); whole product—leaves and petioles (celeriac leaves, Swiss chard, spinach); whole product after removal of stem and calyx lobes (aubergine, butternut, cucumber, melon, pepper, tomato, watermelon, zucchini); whole product (bean, green bean, peas); whole product after removal of top and soil (beetroot, carrot, celeriac, chicory root, horseradish, parsley root, potato, radish, sweet potato); whole plant after removal of root and decayed leaves (broccoli, cabbage, cauliflower, Chinese cabbage, radicchio, savoy cabbage); only cabbage buttons (Brussels sprout); dried product whole, ground (fennel, ginger); mature bulbs after removal of easily detachable skin and soil (garlic, onion); whole product after removal of root, top and soil (kohlrabi); whole product after removal of soil/growing medium (mushroom); kernel with shell (poppy seed, sesame seed); brown rice after removal of the hull (rice); kernel (wheat) [10,12,13,14,15]. Thus, in addition to vegetables, analyses were also conducted on other plant species: sugar plant (chicory root), spices (fennel, ginger), oilseeds (poppy seed, sesame seed), and cereals (rice, wheat).

### 2.2. Chemicals and Standards

Acetonitrile (ACN) of LC/GC-MS grade was purchased from PanReac (Darmstadt, Germany) and used as an extraction solvent. Ammonium formate and formic acid of LC-MS grade were purchased from Fisher Chemical (Pittsburgh, PA, USA). All QuEChERS mixes are purchased from Macherey-Nagel (Düren, Germany). The QuEChERS Mix I (mixture of 4 g magnesium sulphate, 1 g sodium chloride, 0.5 g disodium hydrogen citrate sesquihydrate, and 1 g sodium citrate dihydrate), QuEChERS Mix III (mixture of 900 mg MgSO_4_, and 150 mg CHROMABOND Diamino), QuEChERS Mix IV (mixture of 900 mg MgSO_4_, 150 mg CHROMABOND Diamino, and 15 mg CHROMABOND Carbon), and QuEChERS Mix VI (mixture of 900 mg MgSO_4_, 150 mg CHROMABOND Diamino, and 150 mg CHROMABOND C_18_). Triphenyl phosphate (TPP), used as an internal standard, was purchased from Dr. Ehrenstorfer GmbH (Augsburg, Germany). The mobile phase consisted of 0.1% formic acid and 4 mM ammonium formate in water (MP-A) and 0.1% formic acid and 4 mM ammonium formate in methanol (MP-B). All reagents and standards were freshly prepared in LC-MS grade water or solvent prior to use. The list of the 148 analyzed pesticides and the quality control programme is provided in Supplementary Material S1.

### 2.3. Stock and Working Solutions

Individual pesticide standards for all analytes were purchased from Dr. Ehrenstorfer GmbH (Germany). A working pesticide solution with a concentration of 1 mg/L was prepared as a mixture of individual pesticide standards with stock concentrations of approximately 1000 mg/L, depending on their purity, by transferring 10 µL of each individual stock solution into a 10 mL volumetric flask and diluting to volume with ACN. The working pesticide solution was prepared after every 100 injections. Individual stock standard solutions (1000 mg/L) were stored in the dark at –18 °C and used for no longer than 6 months. The internal standard stock solution (1000 mg/L) of TPP was prepared by dissolving 10 mg of TPP in a 10 mL volumetric flask and diluting to volume with ACN. The working internal standard solution (10 mg/L) in ACN was also prepared for matrix spiking.

### 2.4. Preparation of Samples and Control Samples

Samples were prepared in the following procedure. After grinding and homogenization (knife mill GRINDOMIX GM 200, Retsch, Germany), 5 g of rice sample and 10 g of cabbage sample, separately, were measured into 50 mL polypropylene centrifuge tubes (PP tube). A total of 100 μL of internal standard and an appropriate solution of stock pesticide standard were added so that the final concentrations were 50 μg/kg of TPP for both matrixes and 10 mL of cold water was added to the tube with rice and 10 mL of extraction solvent (ACN) was added in both samples. After homogenization by hand shake (1 min), QuEChERS Mix I was added. The whole content of the tube was hand shaken for another 1 min and centrifuged for 5 min at 5000 rpm. For the determination of pesticides by the LC technique, 500 μL of the extract was transferred into a vial, and 500 μL of MP-A and 10 μL of 5% formic acid in ACN were added. For the determination of pesticides by the GC technique, 6 mL of the extract was transferred into a clean-up tube, followed by the addition of the appropriate QuEChERS Mix (QuEChERS Mix III, QuEChERS Mix IV, and QuEChERS Mix VI) according to the recommendation of the QuEChERS manufacturer Macherey-Nagel (Germany) [103]. The whole content of the tube was hand shaken for another 30 s and centrifuged for 5 min at 5000 rpm. Additionally, 500 μL of the extract was transferred into a vial, and 500 μL of toluene (PanReac, Germany) and 10 μL of 5% formic acid in ACN were added. Prior to the analysis all extracts were filtered through 0.45 μm polytetrafluoroethylene (PTFE) filters.

The control spike samples were prepared in the following procedure. After grinding and homogenization (knife mill GRINDOMIX GM 200, Retsch, Germany), 5 g of rice sample and 10 g of cabbage sample were separately measured into 50 mL polypropylene centrifuge tubes (PP tube). A total of 100 μL of internal standard and 100 μL of stock pesticide standard solutions (for each analyte separately) were added so that the final concentrations were 10 and 100 μg/kg for rice and 5 and 50 μg/kg for cabbage samples. A total of 10 mL of cold water was added to the tube with rice and 10 mL of extraction solvent (ACN) was added to both samples. After homogenization by hand shake (1 min), QuEChERS Mix I was added. The whole content of the tube was hand shaken for another 1 min and centrifuged for 5 min at 5000 rpm. For the determination of pesticides by the LC technique, 500 μL of the extract was transferred into a vial, and 500 μL of MP-A and 10 μL of 5% formic acid in ACN were added. For the determination of pesticides by the GC technique, 6 mL of the extract was transferred into a clean-up tube, followed by the addition of the appropriate QuEChERS Mix (QuEChERS Mix III, QuEChERS Mix IV, and QuEChERS Mix VI). The whole content of the tube was hand shaken for another 30 s and centrifuged for 5 min at 5000 rpm. A total of 500 μL of the extract was transferred into a vial, and 500 μL of toluene (PanReac, Germany) and 10 μL of 5% formic acid in ACN were added. Prior to the analysis all extracts were filtered through 0.45 μm polytetrafluoroethylene (PTFE) filters.

Matrix-matched calibration standards were prepared as follows. After grinding and homogenization, 5 g of the rice sample and 10 g of the cabbage sample were separately weighed into a 50 mL PP tube, and 10 mL of cold water was added to the tube with the rice. A total of 10 mL of extraction solvent (ACN) and 100 μL of internal standard solution (10 mg/L) were added to each suspension. After homogenization by hand shake (1 min), QuEChERS Mix I was added, whereas an additional clean-up step using QuEChERS Mix III, IV, or VI was applied for GC analysis, depending on the matrix. The whole content of the tube was hand shaken for another 1 min and centrifuged for 5 min at 5000 rpm. In the final step of calibration standard sample preparation, a defined volume of the working mixed pesticide standard solution (1 mg/mL) was added to obtain final concentrations of 5, 10, 20, 50, 100, 200, and 400 μg/kg for rice samples, and 10, 20, 40, 100, 200, 400, and 800 μg/kg for cabbage samples using appropriate volumes of standard stock solution (1 mg/L) and matrix match sample extract to 500 µL and 500 µL MP-A (for LC analysis)/toluene (for GC analysis). Finally, 10 μL of 5% formic acid in ACN was added to each vial. Blank samples for matrix match calibration were prepared as described without pesticide standards. Prior to the analysis, all extracts were filtered through 0.45 μm PTFE filters.

### 2.5. Matrix Effect

Matrix blank samples, which are purchased at the local market for high-quality products. All matrices were checked for pesticide residues prior to usage as matrix blanks. Matrix effect was investigated by two matrix-matched standard levels, one without dilution (total volume 1 mL) and one diluted (500 μL of extract diluted with 500 μL of MP-A). The matrix effect was evaluated using two vegetable matrices, while additional details are provided in the Supplementary Material S1. Matrix-matched standards were compared to standard solutions of the same analytes (ACN:MP-A = 1:1) and the ME (%) was calculated according to formula [104]:

$$ME=(\frac{slope\;of\;matrix\;matched\;standard}{slope\;of\;standard\;solution}-1)\cdot 100$$

According to literature [105], it is mild or tolerable when ME equals to −20–+20%, medium with ME more than ±20% up to ±50%, and strong when ME is more than ±50%.

### 2.6. Instruments and Chromatographic Conditions

Pesticide residues in this study were determined using two chromatographic techniques: liquid chromatography (LC) and gas chromatography (GC), coupled with tandem mass spectrometry (LC-MS/MS and GC-MS/MS). A total of 113 pesticide residues were determined using LC-MS/MS, while 35 pesticide residues were determined using GC-MS/MS (Supplementary Material S1).

LC analysis: Pesticides were analyzed using a Thermo Scientific UHPLC (Waltham, MA, USA) (ultra-high-performance liquid chromatography) system, Dionex UltiMate 3000, coupled with an MS detector, triple-quadrupole Thermo Scientific TSQ Quantum Access Max with electrospray ionization. The mass spectrometer was operated in positive mode and its parameters were spray voltage 5.0 kV, vaporizer temperature 280 °C, ion transfer tube temperature 300 °C, auxiliary gas (nitrogen) pressure 1.81 L/min, and sheath gas pressure 6.64 L/min. Collision gas was argon (pressure set at 1.5 mTor). The selected reaction monitoring (SRM-EZ) method was applied. Chromatographic separation was carried out with reverse-phase Thermo Scientific Hypersil Gold aQ (100 mm 2.1 mm, i.d. 1.9 μm) analytical column, under the following gradient profile: 0–2.1 min, 95% MP-A; 2.1–25.0 min, 95-5% MP-A; 25.0–35.0 min, 5% MP-A; 35.0–35.1 min, 5–95% MP-A; 35.1–40.8 min, 95% MP-A. The flow rate of the mobile phase was 0.3 mL/min, the injection volume was 6 μL and the column oven temperature was 40 °C.

GC Analysis: Pesticides were also analyzed using a gas chromatography–tandem mass spectrometry system consisting of a Thermo Scientific Trace 1300 gas chromatograph coupled to a TSQ 9000 triple quadrupole mass spectrometer. Chromatographic separation was achieved using a TG-5SILMS capillary column (30 m × 0.25 mm i.d., 0.25 μm film thickness; Thermo Scientific, Waltham, MA, USA). The injection volume was 1.2 μL, using a splitless injection mode with an SSL single taper liner. The inlet temperature was set to 90 °C. Helium was used as the carrier gas at a constant flow rate of 1.2 mL/min. The oven temperature programme was as follows: an initial temperature of 70 °C (held for 1.5 min), increased to 90 °C at 25 °C min^−1^ (held for 1.5 min), then raised to 180 °C at 25 °C/min (no hold), followed by an increase to 280 °C at 5 °C/min (no hold), and finally to 300 °C at 10 °C/min, with a final hold time of 5 min. The mass spectrometer operated in electron ionization (EI) mode at 70 eV. The ion source temperature was maintained at 300 °C, while the transfer line temperature was set to 280 °C. Data acquisition was performed in tandem mass spectrometry (MS/MS) mode. Argon was used as the collision gas at a pressure of 60 psi in Q2. The quadrupole resolution was set to 0.8 Da for both Q1 and Q3.

Data acquisition and processing were performed using TraceFinder General Quan 3.2 and Xcalibur 3.0 software for LC analysis, and TraceFinder EFS 5.1 SP3 and Xcalibur 4.5 SR1 for GC analysis. The LC acquisition method was MULTI_PESTICIDES_LC, while the GC acquisition method was MULTI_PESTICIDES_GC.

### 2.7. Validation Parameters

Linearity, precision (RSDr), accuracy (recovery), specificity, limit of detection, and limit of quantification were analytical parameters evaluated according to Document SANTE/12682/2019 (Document No SANTE/12682/2019 Method Validation & Quality Control Procedures for Pesticide Residues Analysis in Food & Feed, n.d.) [106].

Linearity was evaluated for each analyte and each matrix through matrix-matched calibration at at least 5 points. Experimentally determined linear ranges were 10–800 μg/kg for rice and 5–400 μg/kg for cabbage. For each pesticide, correlation coefficients were r^2^ ≥ 0,99, with residual values lower than 20% for each calibration point. Calibration curves obtained for internal standard addition and external standards were evaluated. Calibration by external standards was acceptable for the majority of pesticides. However, for quantification purposes the internal standard method was used. The limit of detection (LOD) was calculated as 3.3σ/s, where σ is the standard deviation (5 control spiked samples) and *s* is the slope of the calibration curve, and the limit of quantification (LOQ) was calculated as 10σ/s.

The concentration of control spiked samples for LOD and LOQ evaluation was 0.005 mg/kg and 0.01 mg/kg in cabbage and rice respectively. For the evaluation of precision and accuracy, higher and lower spiking concentrations were used for each matrix. The lower concentrations were 0.005 mg/kg and 0.01 mg/kg, and the higher concentrations were 0.05 mg/kg and 0.1 mg/kg in cabbage and rice respectively. Five control spiked samples were used. Precision (RSD, %) and accuracy (Recovery, %) for each series were calculated.

For cabbage, average recoveries were 70–117% for a concentration level of 5 µg/kg and 70–115% for a concentration level of 50 µg/kg, with precision RSDr < 17%. The limits of detection and limit of quantification for cabbage were in ranges of 0.4–6.3 and 1.2–19 µg/kg, respectively. For rice, average recoveries were 70–110% for a concentration level of 10 µg/kg and 70–115% for a concentration level of 100 µg/kg with precision RSDr < 15%. The limits of detection and quantification for rice were in the ranges of 0.5–5.5 and 1.5–16.8 µg/kg, respectively.

The results were evaluated according to the reporting limit (RL) and MRLs established by Serbian regulation. The reporting limit (RL) was 0.01 mg/kg. Generally, Serbian, as well as EU, MRLs for pesticide residues in vegetables are in the range of 0.01–10 mg/kg, depending on the compound. Only for a few pesticides are MRLs up to 15 and 20 mg/kg.

## 3. Results and Discussion

Detailed characteristics like common name, country of origin, and number of samples (without and with pesticide residues) of the analyzed samples are shown in Table 1. All pesticide residues at or above the reporting limit (RL ≥ 0.01 mg/kg) are presented.

**Figure 1:**
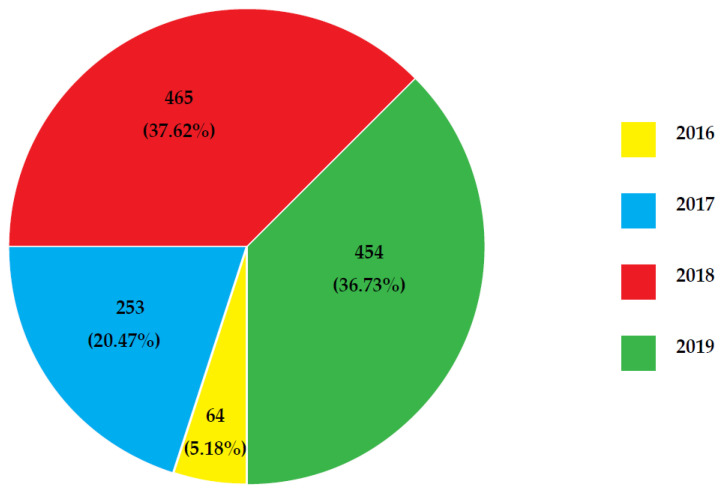
Number and percentage of samples analyzed in 2016, 2017, 2018, and 2019.

**Figure 2:**
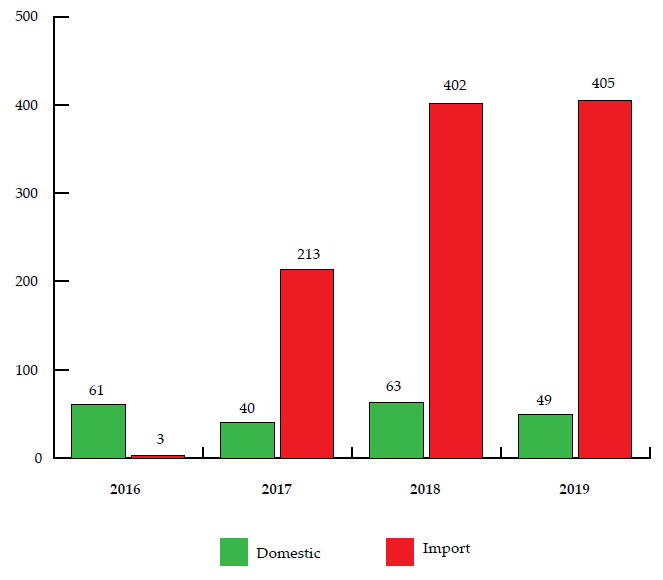
Number of samples of different origins analyzed in 2016, 2017, 2018, and 2019.

In this study, a total of 1236 samples of fresh vegetables were analyzed for pesticide residue. The number and percentage of samples collected per year are shown in Figure 1. The origin of the samples is shown in Figure 2. Evaluation of the obtained results for the 1236 different vegetable samples (Table 1) showed that 40.13% (496 out of 1236 samples) contained pesticide residues (RL ≥ 0.01 mg/kg), while 59.87% (740 out of 1236 samples) contained no pesticide residues (RL < 0.01 mg/kg). All samples of butternut squash (*n* = 3), fennel (*n* = 2), kohlrabi (*n* = 1), poppy seed (*n* = 1), radicchio (*n* = 3), fig (*n* = 1), savoy cabbage (*n* = 1), sweet potato (*n* = 5), and wheat (*n* = 1) were pesticide-free. The detection rates (when the sample size “*n*” is greater than 30) of pesticide residues in aubergine (*n* = 42), bean (*n* = 64), cabbage (*n* = 48), celeriac (*n* = 35), cucumber (*n* = 91), lettuce (*n* = 52), onion (*n* = 87), pepper (*n* = 124), potato (*n* = 159), spinach (*n* = 41), tomato (*n* = 187), and watermelon (*n* = 47) samples were 38.10%, 26.56%, 16.67%, 57.14%, 52.74%, 59.62%, 12.64%, 45.97%, 43.40%, 53.66%, 46.52%, and 25.53%, respectively. All pesticide residues detected in arugula (*n* = 10), bean (*n* = 64), beetroot (*n* = 10), broccoli (*n* = 14), Brussels sprout (*n* = 6), butternut (*n* = 3), cabbage (*n* = 48), carrot (*n* = 23), cauliflower (*n* = 25), chicory root (*n* = 1), Chinese cabbage (*n* = 2), cornsalad (*n* = 2), fennel (*n* = 2), garlic (*n* = 17), green bean (*n* = 4), horseradish (*n* = 2), kohlrabi (*n* = 1), leek (*n* = 12), lettuce (*n* = 52), melon (*n* = 16), mushroom (*n* = 2), onion (*n* = 87), parsley root (*n* = 3), peas (*n* = 6), poppy seed (*n* = 1), radicchio (*n* = 3), radish (*n* = 8), rice (*n* = 22), savoy cabbage (*n* = 1), sweet potato (*n* = 5), watermelon (*n* = 47), wheat (*n* = 1), and zucchini (*n* = 23) were below or at the MRLs. The MRLs for pesticide residues were exceeded in 22 out of the 1236 (1.78%) samples: aubergine (one out of the 42 samples, 2.38%; Spain), celeriac (one out of the 35 samples, 2.86%; Serbia), celeriac leaves (one out of the five samples, 20.00%; The Netherlands), cucumber (three out of the 91 samples, 3.30%; Albania: *n* = 2, Serbia: *n* = 1), ginger (five out of the 23 samples, 7.22%; Spain: *n* = 2, Swaziland: *n* = 1, Turkey: *n* = 11), pepper (two out of the 124 samples, 1.61%; North Macedonia, Serbia), potato (one out of the 159 samples, 0.63%; Germany), sesame seed (two out of the five samples, 40.00%; India), spinach (three out of the 41 samples, 7.32%; Italy: *n* = 2, Poland: *n* = 1), and tomato (two out of the 187 samples, 1.07%; Albania, Italy).

The frequency of the detected pesticide residues in 1236 vegetable samples is shown in Table 2. A total of 148 pesticide residues (distributed as follows: 47.30% insecticides, 37.84% fungicides, and 14.86% herbicides) were detected in all the vegetable samples.

Imidacloprid, boscalid, acetamiprid and prothioconazole, methoxyfenozide, imazalil and propamocarb, metalaxyl and thiacloprid, spiromesifen, amitraz and propham, fludioxonil, and thiophanate-methyl were the pesticide residues most frequently found (occurrence in more than 300 analyzed samples, >24%) and were detected in 74 (5.99%), 61 (4.94%), 37 (2.99%), 34 (2.75%), 30 (2.43%), 29 (2.35%), 28 (2.27%), 24 (1.94%), 22 (1.78%), and 20 (1.62%) samples, respectively. Of the 148 pesticide residues, 22 (14.86%) were detected at least once in vegetable samples at levels higher than MRLs. A total of 30 pesticide residues (nine in ginger, 30.00%; four each in cucumber, spinach, and tomato, 13.33%; two each in papper and sesame seed, 6.66%; 1 each in aubegrine, celeriac, celeriac leaves, potato, and Swiss chard) were found in the 22 vegetable samples containing residues above MRLs. In 2016, one sample with pesticide residue content higher than the MRL was identified; in 2017, two samples; in 2018, eleven samples; and in 2019, eight samples. The other 162 (88.04%) pesticide residues did not exceed their MRL values. The most frequent pesticide residues found to exceed the MRL were carbofuran, formetanate, phoxim, procymidone, tebufenpyrad, and zoxamide (100%, one out of one sample), oxamyl (50.00%, one out of two samples), etoxazole, formothion, malaoxon, and pirimiphos-methyl (33.33%, one out of three samples), chlothianidin (23.08%, three out of 13 samples), chlorpyrifos and methamidophos (14.29%, two out of 14 samples, and one out of seven samples, respectively), (14.39%, two out of 14 samples), and triadimenol (12.50%, one out of eight samples).

Pesticide residues in vegetable samples were reported in many countries. Table 3 presents the available results on pesticide residues in vegetables where the levels exceeded the MRLs and where the number of investigated samples was equal to or higher than 50, using a multi-residue analytical method.

Of the 22 pesticide residues identified in this study with levels exceeding the MRL, nine were also reported in other studies and countries, as presented in Table 3. Chlorpyrifos levels exceeding the MRL were reported in 21 studies [23,25,27,28,29,31,32,37,38,54,57,58,61,67,75,77,78,82,84,86,89]; Methamidophos in 9 studies [23,25,26,27,29,54,57,58,90]; Carbendazim in 7 studies [18,22,25,27,29,39,58]; Acetamiprid [26,28,38,39,56], Carbofuran [30,56,67,75,82], and Procymidone [29,33,38,61,83] in 5 studies each; Methomyl in 3 studies [26,29,34]; Phoxim in 2 studies [38,39]; and Prothioconazole in one study [29]. Consistent with the results of the present study, chlorpyrifos residues exceeding the maximum residue limits (MRLs) in cucumbers and tomatoes were reported by El-Mageed et al. [28], Chowdhury et al. [67], and Łozowicka et al. [77]. Exceedances exclusively in cucumbers were reported by Ibrahim et al. [29] and Osaili et al. [58], whereas exceedances exclusively in tomatoes were reported by Calderon et al. [23], Omwenga et al. [57] and Latif et al. [75]. Methamidophos residues exceeding the MRL in peppers (chilli and sweet peppers) were reported by Ibrahim et al. [29], Blankson et al. [54], Osaili et al. [58], and Yu et al. [90]. Carbofuran residues exceeding the MRL in celeriac leaves were reported by Jiang et al. [30]. Procymidone residues exceeding the MRL in bulbs were reported by Tong et al. [38], while methomyl residues exceeding the MRL in peppers were reported by Ibrahim et al. [29].

In addition, pesticide residues above the MRLs were detected in several countries when vegetables and fruits were tested simultaneously. Pesticide residues exceeding the MRLs were detected in vegetables and fruits in the following studies: Algharibeh and AlFararjeh [41] in Jordan (*n* = 158; 22%), Gondo et al. [42] in Botswana (*n* = 83; 13%), Hjorth et al. [43] in Nordic countries (*n* = 724; 8.4%; vegetables and fruits from South America—a Nordic project, monitoring programme), Ibrahim et al. [44] in Egypt (*n* = 175; 42%), Jallow et al. [45] in Kuwait (*n* = 150; 21%), Mac Loughlin et al. [46] in Argentina (*n* = 135; 36.3%), Mebdoua et al. [47] in Algeria (*n* = 160; 12.5%), Mutengwe et al. [48] in South Africa (N = 53; 1.9%), Poulsen et al. [49] in Denmark [*n* = 17309— vegetable and fruit (70%); 2.6%, monitoring programme], Shin et al. [50] in South Korea (*n* = 115; 0.87%), Skretteberg et al. [51] in Nordic countries (*n* = 721; 12%; vegetables and fruits from Southeast Asia—a Nordic project, monitoring programme), Soydan et al. [52] in Turkey (*n* = 3044; 11.6%), Toptanci et al. [53] in Turkey (*n* = 493; 29.2%), Jardim and Caldas in Brazil [62] (*n* = 13,556; 2.7%; Brazilian monitoring programme), Mert et al. [63] in the UK (*n* = 25,822; 4.0%; monitoring programme), Park et al. [64] in Korea (*n* = 1146; 1.0%), Al-Shamary et al. [92] in Qatar (*n* = 127; 62.2%), Bempah et al. [93] in Ghana (*n* = 350; 19.0%), Chen et al. [94] in China (*n* = 3009; 11.7%; monitoring programme), Knežević et al. [95] in Croatia (*n* = 866; 5.3%), Luo et al. [96] in China (*n* = 3307; 1.0%), Mutengwe et al. [97] in South Africa (*n* = 199; 1.0%), Mutengwe et al. [98] in South Africa (*n* = 37,838; 0.32%; monitoring programme), Patiño et al. [99] in Colombia (*n* = 100; 41.0%), Sivaperumal et al. [100] in India (*n* = 286; 16.4%), and Szpyrka et al. [101] in Poland (*n* = 1026; 1.8%). Thus, the percentage of pesticide residues in vegetable and fruit samples exceeding the MRL ranged from 0.32% to 62.2% across various studies. Moreover, the use of banned/prohibited/withdrawn/not-approved/non-authorized/restricted/non-registered/non-recommended active ingredients was identified in vegetables and fruits from many different countries [19,27,28,30,37,38,39,41,42,45,46,48,56,58,61,62,66,71,74,75,77,79,82,83,86,88,89,90,91,97,98,99,101].

In order to enhance the level of food safety for the benefit of consumer protection, the European Union established the Rapid Alert System for Food and Feed (RASFF) in 197. Members of the network use RASFF to notify each other about dangerous health hazards such as heavy metals, pathogenic microorganisms, and pesticide residues. Moreover, RASFF reported pesticides as the third most widely known hazard category [6]. For the same reason, EFSA publishes an annual report on pesticide residues in all food commodities in the European Union every year, based on the results of a surveillance programme involving all member states [107,108,109,110].

An overview of the number of residues per sample in our research is shown in Table 4.

Many samples contained several pesticide residues. A total of 1083 individual pesticide residues were found in the 496 vegetable samples containing residues. Of the 1236 samples analyzed, a single pesticide residue was detected in 224 (18.12%) samples, while two, three, four, five, and six pesticide residues were detected in 132 (10.68%), 52 (4.21%), 43 (3.48%), 21 (1.70%), and 15 (1.21%) samples, respectively. Seven or more pesticide residues were detected in 0.73% of the samples. The highest number of pesticide residues was found in the tomato sample, containing 10 pesticide residues.

In the previously cited studies, multiple pesticide residues (more than two pesticides per sample) were found in most vegetable samples: 55.6% in eggplants and tomatoes from Sudan [17], 32.8% in tomatoes from Colombia [18], 29.6% in vegetables from Italy [19], 20.0% in vegetables and fruits from Turkey [20], 56% in eggplants, 96% in chilli and in all tomatoes from Nepal [22], 22% in vegetables from Ethiopia [24], 7.5% in ready-to-eat leafy vegetables from Chile [25], 22.5% in tomatoes and lettuces from Chile [26], 65.3% in leafy vegetables from Chile [27], 12.5% in wheat grains and its products from Algeria [32], 32.5, 3.6, 1.33, 10.9, and 6.0% in vegetables from China [33,60,76,84,89], 55.9% in vegetables from Saudi Arabia [34], 26.0% in leafy green vegetables from Italy [35], 34.1% in vegetables from India [37], 15.0% in minor vegetables from China [39], 59.5% in cowpeas from China [40], 17% in French beans and tomatoes from Kenya [57], 1.55% in leafy vegetables, stalk and stem vegetables from South Korea [59], 21.9% in vegetables from Korea [61], 10.5% in vegetables from Bangladesh [67,73], 25% in vegetables from Vietnam [70], 30% in tomatoes and cucumbers from Kazakhstan [77], 4% in *Brassica* vegetables from Poland [78], and 12.9% in vegetables from Thailand [86]. In addition, multiple pesticide residues were also found in vegetables in many other previously cited studies, but the percentages were not clearly stated, or rather, not calculated.

The occurrence of multiple pesticide residues in a single sample may indicate the simultaneous or sequential application of different plant protection products during agricultural production. The presence of multiple residues can increase concern regarding potential combined or cumulative effects on human health, particularly in cases of long-term dietary exposure. Compounds most frequently detected at concentrations exceeding the maximum residue levels (MRLs) may suggest inappropriate pesticide application practices, such as excessive dosage, non-compliance with pre-harvest intervals, or misuse of registered products.

MRLs are established primarily as regulatory limits based on good agricultural practice and are used to monitor compliance in food production and trade. Exceeding an MRL does not necessarily indicate an immediate health risk to consumers; however, it may indicate the need for further toxicological evaluation and dietary exposure assessment, especially when repeated exceedances or multiple residues are observed. Continuous monitoring of pesticide residues is therefore essential to ensure food safety and consumer protection.

Furthermore, continued research and additional data collection on pesticide residues in vegetables will enable more comprehensive dietary exposure assessments and a better evaluation of the potential cumulative health risks associated with pesticide intake.

### Study Limitations and Future Perspectives

This study provides important data on pesticide residue concentrations in vegetables; however, several limitations should be considered. Although a relatively large number of samples collected within an official monitoring programme were analyzed over a four-year period, the sample set was limited to vegetables submitted for official control at the request of the Ministry of Agriculture, Forestry and Water Economy of the Republic of Serbia in an accredited laboratory prior to market placement and therefore may not fully represent all vegetable products or all exposure scenarios. In addition, not all pesticide compounds were included in the analysis, and seasonal variations in residue levels were not assessed.

Dietary exposure assessment and cumulative risk evaluation were not performed due to the lack of reliable data on the consumption quantities of individual vegetable types, which are essential for such analyses. The present study also did not include information on food preparation and processing effects.

Future research should therefore include continued monitoring and the collection of more representative datasets within monitoring programmes, covering a broader range of pesticides, vegetable types, and seasonal variations. Furthermore, the inclusion of dietary exposure assessment, cumulative risk analysis, and the impact of food processing would provide a more comprehensive evaluation of potential health risks associated with pesticide residue concentrations.

## 4. Conclusions

In this study, pesticide residues were investigated in 1236 samples of commonly consumed vegetables in Serbia collected between 2016 and 2019. The results indicate that although a considerable proportion of samples (40.13%; *n* = 496) contained detectable pesticide residues, only a small fraction (1.78%; *n* = 496) exceeded the established MRLs, suggesting a generally high level of compliance with regulatory standards.

The occurrence of MRL exceedances, particularly in specific commodities such as ginger (a spice product), cucumber (a fruiting vegetable), and spinach (a leafy vegetable), highlights the importance of targeted monitoring of high-risk vegetables and plant products. In addition, the presence of multiple residues in certain samples suggests the need for further attention to combined exposure and potential cumulative effects.

Overall, the findings demonstrate that pesticide contamination in vegetables is present but generally within acceptable regulatory limits. Nevertheless, the observed exceedances underline the need for continuous monitoring programmes to ensure food safety and protect public health. Future studies should further include dietary exposure and cumulative risk assessments to provide a more comprehensive evaluation of potential health risks associated with pesticide intake.

When compared with previously published data on fruits [16], vegetables showed a lower overall frequency of pesticide contamination, indicating possible differences in pesticide usage patterns and residue behaviour between these food groups, with mean residue level (MRL) exceedance rates of 4.67% in fruits and 1.78% in vegetables.

## Figures and Tables

**Table 1:** Characteristics of the analyzed vegetable samples and the number of vegetable samples without and with pesticide residues.

Name of the Vegetable Samples	Country of Origin	No. of Samples	No. of Samples Without Residues (<0.01 mg/kg)	%	No. of Samples with Residues at or Above 0.01 mg/kg	%	No. of Samples with Residues Above the MRL	%
Arugula	Italy (*n* = 7), Serbia (*n* = 3)	10	1	10.00	9	90.00	0	0
Aubergine	Albania (*n* = 1), Greece (*n* = 12), Italy (*n* = 5), North Macedonia (*n* = 3), Serbia (*n* = 6), Spain (*n* = 9), The Netherlands (*n* = 6)	42	26	61.90	16	38.10	1	2.38
Bean	Albania (*n* = 1), Argentina (*n* = 7), Bulgaria (*n* = 3), Canada (*n* = 6), Egypt (*n* = 8), France (*n* = 1), Italy (*n* = 1), Kazakhstan (*n* = 3), Kyrgyzstan (*n* = 13), Poland (*n* = 15), Serbia (*n* = 6)	64	47	73.44	17	26.56	0	0
Beetroot	Poland (*n* = 4), Serbia (*n* = 5), Spain (*n* = 1)	10	9	90.00	1	10.00	0	0
Broccoli	Italy (*n* = 1), Poland (*n* = 2), Serbia (*n* = 3), Spain (*n* = 7), The Netherlands (*n* = 1)	14	10	71.43	4	28.57	0	0
Brussels sprout	Belgium (*n* = 3), The Netherlands (*n* = 1)	6	2	33.33	4	66.66	0	0
Butternut squash	Brazil (*n* = 1), Portugal (*n* = 1), Spain (*n* = 1)	3	3	100	0	0	0	0
Cabbage	Albania (*n* = 3), Egypt (*n* = 1), North Macedonia (*n* = 19), Poland (*n* = 1), Portugal (*n* = 1), Serbia (*n* = 17), The Netherlands (*n* = 6)	48	40	83.33	8	16.67	0	0
Carrot	Austria (*n* = 1), Italy (*n* = 7), Serbia (*n* = 17), The Netherlands (*n* = 4)	23	9	39.13	14	60.87	0	0
Cauliflower	Albania (*n* = 1), France (*n* = 5), Germany (*n* = 1), Italy (*n* = 7), North Macedonia (*n* = 1), Poland (*n* = 2), Serbia (*n* = 4), Spain (*n* = 1), The Netherlands (*n* = 3)	25	20	80.00	5	20.00	0	0
Celeriac	Poland (*n* = 2), Serbia (*n* = 10), The Netherlands (*n* = 23)	35	15	42.86	20	57.14	1	2.86
Celeriac leaves	Belgium (*n* = 1), Great Britain (*n* = 1), The Netherlands (*n* = 3)	5	0	0	5	100	1	20.00
Chicory root	Tunisia (*n* = 1)	1	0	0	1	100	0	0
Chinese cabbage	Poland (*n* = 1), The Netherlands (*n* = 1)	2	0	0	2	100	0	0
Cornsalad	Italy (*n* = 2)	2	0	0	2	100	0	0
Cucumber	Albania (*n* = 31), Croatia (*n* = 9), Greece (*n* = 5), Italy (*n* = 2), North Macedonia (*n* = 9), Serbia (*n* = 21), Spain (*n* = 11), The Netherlands (*n* = 2), Turkey (*n* = 1)	91	43	47.25	48	52.75	3	3.30
Fennel	Serbia (*n* = 1), The Netherlands (*n* = 1)	2	2	100	0	0	0	0
Garlic	Austria (*n* = 1), China (*n* = 11), Egypt (*n* = 1), Iran (*n* = 1), Italy (*n* = 1), Spain (*n* = 1)	17	12	70.59	5	29.41	0	0
Ginger	Brazil (*n* = 4), China (*n* = 16), Thailand (*n* = 2), The Netherlands (*n* = 1)	23	9	39.13	14	60.87	5	21.74
Green bean	Serbia (*n* = 4)	4	3	75.00	1	25.00	0	0
Horseradish	Hungary (*n* = 2)	2	1	50.00	1	50.00	0	0
Kohlrabi	Italy (*n* = 1)	1	1	100	0	0	0	0
Leek	Albania (*n* = 1), Serbia (*n* = 9), The Netherlands (*n* = 2)	12	8	66.67	4	33.33	0	0
Lettuce	Germany (*n* = 1), Italy (*n* = 6), North Macedonia (*n* = 1), Poland (*n* = 7), Serbia (*n* = 9), Spain (*n* = 15), Tunisia (*n* = 1), The Netherlands (*n* = 11), Turkey (*n* = 1)	52	21	40.38	31	59.62	0	0
Melon	Albania (*n* = 2), Brazil (*n* = 4), Costa Rica (*n* = 2), North Macedonia (*n* = 1), Serbia (*n* = 5), Spain (*n* = 1), South Africa (*n* = 1)	16	7	43.75	9	56.25	0	0
Mushroom	Serbia (*n* = 2)	2	0	0	2	100	0	0
Onion	Albania (*n* = 2), Austria (*n* = 2), Belarus (*n* = 4), Croatia (*n* = 3), Egypt (*n* = 3), France (*n* = 2), Germany (*n* = 5), Italy (*n* = 1), Kazakhstan (*n* = 1), North Macedonia (*n* = 10), Poland (*n* = 4), Russia (*n* = 15), Serbia (*n* = 4), Slovakia (*n* = 1), Spain (*n* = 2), The Netherlands (*n* = 28)	87	76	87.36	11	12.64	0	0
Parsley root	Serbia (*n* = 3)	3	2	66.67	1	33.33	0	0
Peas	Serbia (*n* = 6)	6	3	50.00	3	50.00	0	0
Pepper	Albania (*n* = 17), Bulgaria (*n* = 1), Greece (*n* = 12), Italy (*n* = 3), North Macedonia (*n* = 36), Poland (*n* = 2), Serbia (*n* = 23), Spain (*n* = 17), The Netherlands (*n* = 7), Turkey (*n* = 6)	124	67	54.03	57	45.97	2	1.61
Poppy seed	Turkey (*n* = 1)	1	1	100	0	0	0	0
Potato	Albania (*n* = 1), Austria (*n* = 3), Belarus (*n* = 20), Belgium (*n* = 11), Croatia (*n* = 11), Egypt (*n* = 1), France (*n* = 46), Germany (23), Greece (*n* = 4), Italy (*n* = 7), North Macedonia (*n* = 3), Portugal (*n* = 1), Russia (*n* = 2), Serbia (*n* = 10), Slovenia (*n* = 1), Spain (*n* = 1), The Netherlands (*n* = 13), The United States of America (*n* = 1)	159	90	56.60	69	43.40	1	0.63
Radicchio	Italy (*n* = 2), Poland (*n* = 1)	3	3	100	0	0	0	0
Radish	Italy (*n* = 4), Poland (*n* = 2), Serbia (*n* = 2)	8	4	50.00	4	50.00		0
Rice	Bulgaria (*n* = 1), Greece (*n* = 5), Italy (*n* = 2), North Macedonia (*n* = 13), Thailand (*n* = 1)	22	21	95.45	1	4.55	0	0
Savoy cabbage	Poland (*n* = 1)	1	1	100	0	0	0	0
Sesame seed	China (*n* = 1), Ethiopia (*n* = 1), India (*n* = 3)	5	3	60.00	2	40.00	2	40.00
Spinach	Greece (*n* = 1), Hungary (*n* = 1), Italy (*n* = 22), Poland (*n* = 3), Serbia (*n* = 13), The Netherlands (*n* = 1)	41	19	46.34	22	53.66	3	7.32
Sweet potato	Egypt (*n* = 2), Spain (*n* = 2), The United States of America (*n* = 1)	5	5	100	0	0	0	0
Swiss chard	Serbia (*n* = 1)	1	0	0	1	100	1	100
Tomato	Albania (*n* = 51), Belgium (*n* = 8), Croatia (*n* = 10), Greece (*n* = 14), Hungary (*n* = 1), Italy (*n* = 7), Morocco (*n* = 2), North Macedonia (*n* = 31), Poland (*n* = 3), Serbia (*n* = 18), Spain (*n* = 14), The Netherlands (*n* = 17), Turkey (*n* = 11)	187	100	53.48	87	46.52	2	1.07
Watermelon	Albania (*n* = 2), Greece (*n* = 33), Hungary (*n* = 1), North Macedonia (*n* = 3), Serbia (*n* = 3), Spain (*n* = 3), Turkey (*n* = 2)	47	35	74.47	12	25.53	0	0
Wheat	Serbia (*n* = 1)	1	1	100	0	0	0	0
Zucchini	Albania (*n* = 10), China (*n* = 1), North Macedonia (*n* = 1), Serbia (*n* = 7), Spain (*n* = 1), Turkey (*n* = 3)	23	20	86.96	3	13.04	0	0

**Table 2:** The frequency of the detected pesticide residues and their concentrations in vegetable samples.

Pesticide Name (*n* = 148)	Types of Pesticide	Frequency of Detection in 1236 Samples	%	Name of the Vegetable Samples	No. of Samples with Residues Above MRL	%	Mean ± σ **	Range Min–Max (mg/kg)	MRLs (mg/kg)
2,4-D methyl ester	Herbicide	1	0.08	Tomato	0	0	–	0.010	0.01 (tomato)
2-Phenylphenol	Fungicide	3	0.24	Cucumber, Lettuce	0	0	0.010 ± 0.000	0.010	0.01 (cucumber); 0.1 (lettuce)
Abamectin	Insecticide	1	0.08	Celeriac	0	0	–	0.016	0.05 (celeriac)
Acephate	Insecticide	1	0.08	Pepper	0	0	–	0.010	0.01 (pepper)
Acetamiprid *	Insecticide	37	2.99	Arugula, Aubergine, Celeriac, Cornsalad, Cucumber, Lettuce, Melon, Pepper, Potato, Tomato	1 (celeriac)	2.70	0.043 ± 0.043	0.010–0.203	0.01 (celeriac; potato); 0.1 (potato); 0.2 (aubergine, melon, tomato); 0.3 (cucumber, pepper); 0.5 (tomato); 3 (arugula, cornsalad, lettuce); 5 (arugula, lettuce)
Aclonifen	Herbicide	1	0.08	Celeriac	0	0	–	0.013	0.013 (celeriac)
Aldicarb	Insecticide	8	0.65	Bean, Carrot, Celeriac, Pepper, Tomato, Zucchini	0	0	0.014 ± 0.003	0.010–0.019	0.02 (bean, carrot, pepper, tomato, zucchini); 0.05 (celeriac)
Ametryn	Herbicide	17	1.38	Arugula, Broccoli, Carrot, Cucumber, Onion, Pepper, Potato, Tomato	0	0	0.010 ± 0.000	0.010	0.01 (arugula, broccoli, carrot, cucumber, onion, pepper, potato, tomato)
Aminocarb	Insecticide	2	0.16	Bean, Tomato	0	0	–	0.010	0.01 (bean, tomato)
Amitraz	Insecticide	24	1.94	Aubergine, Bean, Cabbage, Cucumber, Ginger, Lettuce, Onion, Pepper, Spinach, Tomato, Watermelon	1 (tomato)	4.17	0.024 ± 0.034	0.010–0.178	0.05 (aubergine; bean, cabbage, cucumber, ginger, lettuce, onion, pepper, tomato, spinach, watermelon)
Atrazine	Herbicide	8	0.65	Arugula, Broccoli, Cauliflower, Cucumber, Pepper, Swiss chard	0	0	0.023 ± 0.010	0.010–0.040	0.05 (arugula, broccoli, cauliflower, cucumber, pepper, Swiss chard)
Azinphos-methyl	Insecticide	5	0.40	Celeriac, Ginger, Pepper, Swiss chard, Tomato	0	0	0.017 ± 0.009	0.010–0.035	0.05 (celeriac, ginger, pepper, Swiss chard, tomato)
Azoxystrobin	Fungicide	18	1.46	Arugula, Broccoli, Carrot, Chicory root, Cucumber, Lettuce, Peas, Pepper, Tomato	0	0	0.188 ± 0.401	0.010–1.338	0.15 (peas); 1 (carrot, cucumber); 3 (pepper, tomato); 5 (broccoli); 15 (arugula, chicory root, lettuce)
Benalaxyl	Fungicide	1	0.08	Aubergine	0	0	–	0.014	0.5 (aubergine)
Bendiocarb	Insecticide	3	0.24	Melon, Potato, Tomato	0	0	0.010 ± 0.000	0.010	0.01 (melon, potato, tomato)
Beta-cyfluthrin	Insecticide	1	0.08	Cucumber	0	0	–	0.021	0.1 (cucumber)
Bifenazate	Insecticide	10	0.81	Leek, Lettuce, Pepper, Spinach, Tomato	0	0	0.034 ± 0.016	0.020–0.062	0.02 (leek, lettuce, spinach); 0.5 (tomato); 3 (pepper)
Bifenthrin *	Insecticide	6	0.49	Arugula, Celeriac, Cucumber, Pepper	0	0	0.024 ± 0.013	0.010–0.041	0.01 (arugula, cucumber); 0.1 (celeriac, cucumber); 0.5 (pepper)
Biphenyl	Fungicide	1	0.08	Garlic	0	0	–	0.010	0.01 (garlic)
Bitertanol	Fungicide	2	0.16	Arugula, Carrot	0	0	–	0.010	0.01 (carrot); 0.01 (arugula)
Boscalid *	Fungicide	61	4.94	Arugula, Aubergine, Broccoli, Brussels sprouts, Cabbage, Carrot, Celeriac, Cornsalad, Cucumber, Leek, Lettuce, Onion, Pepper, Radish, Spinach, Tomato	0	0	0.101 ± 0.210	0.010–1.221	2 (carrot, celeriac, radish); 3 (aubergine, cucumber, pepper, tomato); 4 (cucumber); 5 (broccoli, Brussels sprouts, cabbage, leek, onion); 30 (arugula, lettuce, spinac,); 50 (arugula, cornsalad, lettuce, spinach)
Carbaryl	Insecticide	1	0.08	Celeriac	0	0	–	0.012	0.05 (celeriac)
Carbendazim	Fungicide	19	1.54	Bean, Cucumber, Ginger, Melon, Mushroom, Tomato, Watermelon	1 (ginger)	5.26	0.083 ± 0.174	0.011–0.763	0.1 (bean, cucumber, ginger, melon, watermelon); 0.3 (tomato); 1 (mushroom)
Carbofuran	Insecticide	1	0.08	Celeriac leaves	1 (celeriac leaves)	100	–	0.108	0.02 (celeriac leaves)
Chlorantraniliprole	Insecticide	19	1.54	Arugula, Aubergine, Cucumber, Ginger, Lettuce, Pepper, Spinach, Tomato	0	0	0.141 ± 0.353	0.010–1.220	0.02 (celeriac leaves); 0.06 (ginger); 0.3 (cucumber); 0.6 (aubergine, tomato); 1 (pepper); 20 (arugula, lettuce, spinach)
Chlorothalonil	Fungicide	2	0.16	Carrot, Tomato	0	0	–	0.010–0.011	0.3 (carrot); 6 (tomato)
Chlorotoluron	Herbicide	1	0.08	Carrot	0	0	–	0.010	0.01 (carrot)
Chlorpropham	Herbicide	19	1.54	Celeriac, Celeriac leaves, Onion, Potato	0	0	0.994 ± 1.564	0.012–5.324	0.02 (celeriac leaves); 0.05 (celeriac, onion); 10 (potato)
Chlorpyrifos	Insecticide	14	1.13	Aubergine, Cabbage, Carrot, Cauliflower, Celeriac, Cucumber, Leek, Mushroom, Pepper, Radish, Tomato	2 (cucumber, tomato)	14.29	0.034 ± 0.028	0.010–0.104	0.01 (cabbage, pepper, tomato); 0.05 (cauliflower, cucumber, leek, mushroom); 0.1 (carrot); 0.2 (radish); 0.5 (aubergine); 5 (celeriac)
Clofentezine	Insecticide	1	0.08	Ginger	0	0	–	0.011	0.02 (ginger)
Clomazone	Herbicide	1	0.08	Celeriac	0	0	–	0.010	0.01 (celeriac)
Clothianidin *	Insecticide	13	1.05	Carrot, Cucumber, Onion, Pepper, Potato, Spinach, Swiss chard, Tomato	3 (spinach, Swiss chard, tomato)	23.08	0.037 ± 0.049	0.010–0.176	0.01 (spinach, Swiss chard); 0.02 (cucumber, onion); 0.03 (potato); 0.04 (tomato); 0.05 (carrot, pepper, potato)
Cyazofamid	Fungicide	2	0.16	Cucumber	0	0	–	0.123–0.132	0.2 (cucumber)
Cyfluthrin	Insecticide	1	0.08	Arugula	0	0	–	0.061	1 (arugula)
Cymoxanil	Fungicide	6	0.49	Celeriac, Cucumber, Tomato	0	0	0.016 ± 0.008	0.010–0.032	0.05 (celeriac); 0.4 (tomato); 0.5 (cucumber)
Cypermethrin	Insecticide	4	0.32	Aubergine, Cabbage, Garlic, Leek	0	0	0.048 ± 0.024	0.014–0.080	0.05 (aubergine, leek); 0.1 (garlic); 1 (cabbage)
Cyproconazole	Fungicide	1	0.08	Parsley root	0	0	–	0.018	0.05 (parsley root)
Cyprodinil *	Fungicide	17	1.38	Arugula, Aubergine, Celeriac, Celeriac leaves, Cucumber, Lettuce, Pepper, Tomato	0	0	0.291 ± 0.951	0.010–4.091	0.1 (lettuce); 0.3 (celeriac); 0.5 (cucumber); 1 (aubergine, tomato); 1.5 (pepper, tomato); 15 (arugula, lettuce); 40 (celeriac leaves)
Deltamethrin	Insecticide	3	0.24	Arugula, Celeriac leaves, Lettuce	0	0	0.010 ± 0.000	0.010	0.01 (arugula, celeriac leaves, lettuce)
Difenoconazole *	Fungicide	17	1.38	Arugula, Beetroot, Carrot, Celeriac, Celeriac leaves, Leek, Tomato	0	0	0.032 ± 0.047	0.010–0.213	0.01 (arugula, carrot, celeriac, leek, tomato); 0.1 (carrot); 0.4 (beetroot); 2 (celeriac, tomato); 10 (celeriac leaves)
Dimethoate	Insecticide	2	0.16	Bean, Mushroom	0	0	–	0.013–0.020	0.02 (bean, mushroom)
Dimethomorph	Fungicide	18	1.46	Arugula, Brussels sprouts, Cucumber, Leek, Lettuce, Melon, Pepper, Radish, Tomato, Zucchini	0	0	0.152 ± 0.429	0.011–1.878	0.01 (Brussels sprouts); 0.5 (cucumber, melon, pepper, zucchini); 1 (radish, tomato); 1.5 (leek), 10 (arugula); 15 (lettuce)
Diniconazole	Fungicide	1	0.08	Potato	0	0	–	0.010	0.01 (potato)
Dinotefuran	Insecticide	4	0.32	Pepper, Potato	0	0	0.010 ± 0.000	0.010	0.01 (pepper, potato)
Emamectin B1a	Insecticide	1	0.08	Arugula	0	0	–	0.012	1 (arugula)
Emamectin B1b	Insecticide	2	0.16	Cucumber, Tomato	0	0	–	0.010–0.011	0.01 (cucumber); 0.02 (tomato)
Epoxiconazole	Fungicide	2	0.16	Horseradish, Potato	0	0	–	0.010–0.024	0.05 (horseradish, potato)
Eprinomectin	Insecticide	1	0.08	Lettuce	0	0	–	0.010	0.01 (lettuce)
Ethiofencarb	Insecticide	1	0.08	Potato	0	0	–	0.010	0.01 (potato)
Ethofumesate	Herbicide	4	0.32	Onion, Pepper, Potato	0	0	0.012 ± 0.002	0.010–0.013	0.05 (onion, pepper, potato)
Etoxazole	Insecticide	3	0.24	Cucumber, Ginger	1 (ginger)	33.33	0.021 ± 0.009	0.010–0.033	0.01 (ginger); 0.02 (cucumber)
Famoxadone	Fungicide	2	0.16	Potato, Tomato	0	0	–	0.014–0.029	0.02 (potato); 1 (tomato)
Fenamidone	Fungicide	2	0.16	Tomato	0	0	–	0.016–0.028	1 (tomato)
Fenarimol	Fungicide	3	0.24	Arugula, Brussels sprouts, Zucchini	0	0	0.014 ± 0.003	0.010–0.018	0.02 (arugula, Brussels sprouts); 0.2 (zucchini)
Fenbuconazole	Fungicide	1	0.08	Ginger	0	0	–	0.026	0.05 (ginger)
Fenhexamid	Fungicide	4	0.32	Bean, Cucumber, Tomato	0	0	0.031 ± 0.016	0.010–0.054	1 (cucumber); 2 (tomato); 5 (bean)
Fenoxycarb	Insecticide	2	0.16	Carrot, Potato	0	0	–	0.010–0.027	0.05 (carrot, potato)
Fenpyroximate	Insecticide	1	0.08	Tomato	0	0	–	0.031	0.2 (tomato)
Fenvalerate	Insecticide	2	0.16	Potato, Swiss chard	0	0	–	0.010	0.02 (potato, Swiss chard)
Flonicamid *	Insecticide	14	1.13	Aubergine, Cucumber, Pepper, Tomato, Watermelon	0	0	0.046 ± 0.033	0.012–0.105	0.15 (pepper); 0.3 (pepper, watermelon); 0.4 (watermelon); 0.5 (aubergine, cucumber, tomato)
Fludioxonil *	Fungicide	22	1.78	Arugula, Cabbage, Celeriac, Cornsalad, Cucumber, Garlic, Lettuce, Pepper, Potato, Swiss chard, Tomato	0	0	0.165 ± 0.350	0.010–1.715	0.05 (garlic); 0,2 (celeriac); 0.4 (cucumber); 1 (pepper, potato); 2 (cabbage, pepper); 3 (tomato); 15 (lettuce); 20 (arugula, cornsalad, Swiss chard); 40 (lettuce)
Fluopicolide	Fungicide	1	0.08	Cucumber	0	0	–	0.017	0.5 (cucumber)
Fluopyram	Fungicide	4	0.32	Aubergine, Cabbage, Cucumber, Pepper	0	0	0.045 ± 0.036	0.011–0.103	0.3 (cabbage); 0.5 (cucumber); 0.9 (aubergine); 2 (pepper)
Fluquinconazole	Fungicide	1	0.08	Tomato	0	0	–	0.014	0.05 (tomato)
Flutolanil	Fungicide	4	0.32	Potato, Spinach, Tomato	0	0	0.017 ± 0.011	0.010–0.035	0.01 (spinach, tomato); 0.5 (potato)
Flutriafol	Fungicide	7	0.57	Pepper	0	0	0.033 ± 0.019	0.014–0.066	1 (pepper)
Formetanate	Insecticide	1	0.08	Cucumber	1 (cucumber)	100	–	0.053	0.01 (cucumber)
Formothion	Insecticide	3	0.24	Garlic, Ginger	1 (ginger)	33.33	0.059 ± 0.069	0.010–0.156	0.01 (garlic, ginger)
Imazalil	Fungicide	30	2.43	Bean, Celeriac, Cucumber, Leek, Melon, Potato, Spinach, Swiss chard, Tomato, Watermelon	0	0	0.032 ± 0.063	0.010–0.332	0.05 (celeriac, leek, spinach, Swiss chard, watermelon); 0.2 (cucumber); 0.5 (tomato); 2 (bean, melon); 3 (potato)
Imidacloprid	Insecticide	74	5.99	Aubergine, Bean, Broccoli, Cabbage, Carrot, Cauliflower, Celeriac, Celeriac leaves, Cucumber, Ginger, Leek, Lettuce, Melon, Onion, Pepper, Potato, Spinach, Tomato, Watermelon	0	0	0.038 ± 0.039	0.010–0.171	0.05 (leek, spinach); 0.1 (onion); 0.2 (watermelon); 0.5 (aubergine, broccoli, cabbage, carrot, cauliflower, celeriac, ginger, melon, potato, tomato); 1 (cucumber, pepper); 2 (bean, celeriac leaves, lettuce)
Indoxacarb	Insecticide	6	0.49	Aubergine, Celeriac, Lettuce, Tomato	0	0	0.021 ± 0.016	0.010–0.055	0.02 (celeriac); 0.5 (aubergine, tomato); 3 (lettuce)
Iprovalicarb	Fungicide	1	0.08	Tomato	0	0	–	0.029	0.7 (tomato)
Kresoxim-methyl	Fungicide	3	0.24	Bean	0	0	0.024 ± 0.004	0.021–0.030	0.05 (bean)
Lambda-cyhalothrin	Insecticide	1	0.08	Arugula	0	0	–	0.024	1 (arugula)
Linuron	Herbicide	8	0.65	Carrot, Celeriac, Tomato	0	0	0.045 ± 0.034	0.011–0.130	0.05 (tomato); 0.2 (carrot); 0.5 (celeriac)
Lufenuron *	Insecticide	14	1.13	Cabbage, Chinese cabbage, Cucumber, Lettuce, Pepper, Potato, Radish, Tomato, Watermelon	0	0	0.018 ± 0.003	0.010–0.021	0.02 (radish); 0.05 (potato); 0.2 (Chinese cabbage, cucumber); 0.3 (watermelon); 0.5 (cabbage, lettuce, tomato); 0.8 (pepper); 1 (pepper)
Malaoxon	Insecticide	3	0.24	Lettuce, Pepper, Spinach	1 (spinach)	33.33	0.087 ± 0.058	0.020–0.162	0.02 (pepper, spinach); 0.5 (lettuce)
Malathion	Insecticide	2	0.16	Cucumber	0	0	–	0.012–0.019	0.02 (cucumber)
Mandipropamid	Fungicide	3	0.24	Arugula, Tomato	0	0	0.909 ± 1.271	0.010–2.706	3 (tomato); 25 (arugula)
Mepanipyrim *	Fungicide	2	0.16	Tomato	0	0	–	0.012–0.032	1 (tomato); 1.5 (tomato)
Mepronil	Fungicide	1	0.08	Pepper	0	0	–	0.010	0.01 (pepper)
Metaflumizone	Insecticide	2	0.16	Tomato	0	0	–	0.014–0.023	0.6 (tomato)
Metalaxyl	Fungicide	29	2.35	Arugula, Celeriac, Cucumber, Lettuce, Melon, Peas, Pepper, Potato, Radish, Spinach, Tomato	0	0	0.033 ± 0.026	0.010–0.130	0.05 (celeriac, peas, potato); 0.1 (radish); 0.2 (melon, tomato); 0.5 (cucumber, pepper); 1.5 (spinach); 3 (arugula, lettuce)
Metalaxyl-M	Fungicide	9	0.73	Celeriac, Cucumber, Ginger, Melon, Peas, Potato	0	0	0.024 ± 0.028	0.010–0.102	0.05 (celeriac, peas, potato); 0.1 (ginger); 0.2 (melon); 0.5 (cucumber)
Methamidophos	Insecticide	7	0.57	Arugula, Pepper, Tomato	1 (pepper)	14.29	0.016 ± 0.014	0.010–0.050	0.01 (arugula, pepper, tomato)
Methiocarb	Insecticide	6	0.49	Aubergine, Broccoli, Pepper, Potato, Tomato, Watermelon	0	0	0.025 ± 0.013	0.011–0.038	0.1 (aubergine, broccoli, potato); 0.2 (pepper, tomato, watermelon)
Methomyl *	Insecticide	13	1.05	Bean, Cucumber, Horseradish, Lettuce, Pepper, Potato, Spinach, Tomato, Zucchini	1 (pepper)	7.69	0.033 ± 0.033	0.011–0.120	0.02 (bean, cucumber, horseradish, pepper, potato, tomato, zucchini); 0.05 (lettuce, spinach); 0.1 (cucumber)
Methoxyfenozide *	Insecticide	34	2.75	Arugula, Aubergine, Cauliflower, Cucumber, Lettuce, Pepper, Potato, Radish, Sesame seed, Spinach, Tomato	1 (potato)	2.94	0.016 ± 0.018	0.010–0.120	0.01 (potato, sesame seed); 0.02 (cauliflower, cucumber, potato); 0.4 (radish); 0.5 (aubergine); 1 (pepper); 2 (tomato); 4 (arugula, lettuce, spinach)
Metobromuron	Herbicide	4	0.32	Lettuce	0	0	0.010 ± 0.000	0.010	0.01 (lettuce)
Metrafenone	Fungicide	1	0.08	Pepper	0	0	–	0.013	2 (pepper)
Metribuzin	Herbicide	8	0.65	Bean, Lettuce, Pepper, Potato, Spinach, Swiss chard, Watermelon	0	0	0.017 ± 0.008	0.010–0.030	0.1 (bean, lettuce, pepper, potato, spinach, Swiss chard, watermelon)
Mevinphos	Insecticide	4	0.32	Leek, Pepper, Potato, Spinach	0	0	0.010 ± 0.000	0.010	0.01 (pepper, spinach, potato, leek)
Myclobutanil	Fungicide	1	0.08	Cucumber	0	0	–	0.012	0.1 (cucumber)
Napropamide	Herbicide	2	0.16	Pepper	0	0	–	0.069–0.085	0.1 (pepper)
Oxamyl	Insecticide	2	0.16	Aubergine	1 (aubergine)	50.00	–	0.020–0.098	0.02 (aubergine)
Penconazole	Fungicide	1	0.08	Cucumber	0	0	–	0.014	0.1 (cucumber)
Pencycuron	Fungicide	5	0.40	Aubergine, Potato	0	0	0.019 ± 0.003	0.014–0.022	0.05 (aubergine); 0.1 (potato)
Permethrin	Insecticide	5	0.40	Ginger	0	0	0.023 ± 0.014	0.012–0.050	0.05 (ginger)
Phenmedipham	Herbicide	1	0.08	Spinach	0	0	–	0.025	0.3 (spinach)
Phosmet	Insecticide	1	0.08	Pepper	0	0	–	0.032	0.05 (pepper)
Phoxim	Insecticide	1	0.08	Sesame seed	1 (sesame seed)	100	–	0.270	0.02 (sesame seed)
Picoxystrobin *	Fungicide	16	1.29	Bean, Cucumber, Parsley root, Pepper, Potato, Radish, Rice, Spinach, Swiss chard, Tomato	0	0	0.019 ± 0.012	0.010–0.050	0.01 (bean, cucumber, parsley root, Swiss chard, tomato); 0.05 (bean, cucumber, pepper, potato, radish, spinach)
Pirimicarb	Insecticide	6	0.49	Cabbage, Lettuce, Spinach, Tomato	0	0	0.025 ± 0.015	0.011–0.057	0.06 (spinach); 0.5 (cabbage, tomato); 1.5 (lettuce)
Pirimiphos-methyl	Insecticide	3	0.24	Bean, Ginger, Spinach	1 (spinach)	33.33	0.026 ± 0.017	0.010–0.049	0.01 (ginger, spinach); 1 (bean)
Prochloraz	Fungicide	2	0.16	Mushroom	0	0	–	0.020–0.024	3 (mushroom)
Procymidone	Fungicide	1	0.08	Tomato	1 (tomato)	100	–	0.321	0.01 (tomato)
Promecarb	Insecticide	1	0.08	Pepper	0	0	–	0.081	3 (pepper)
Prometon	Herbicide	5	0.40	Arugula, Broccoli, Onion, Pepper, Tomato	0	0	0.010 ± 0.000	0.010	0.01 (arugula, broccoli, onion, pepper, tomato)
Prometryn	Herbicide	2	0.16	Cucumber, Pepper	0	0	–	0.010	0.01 (cucumber, pepper)
Propachlor	Herbicide	1	0.08	Celeriac	0	0	–	0.020	0.02 (celeriac)
Propamocarb *	Fungicide	30	2.43	Cucumber, Potato, Spinach, Tomato	0	0	0.140 ± 0.150	0.010–0.576	0.3 (potato); 0.5 (potato); 3 (pepper); 4 (tomato); 5 (cucumber); 10 (cucumber, tomato); 30 (spinach): 40 (spinach)
Propazine	Herbicide	2	0.16	Arugula, Tomato	0	0	–	0.010	0.01 (arugula, tomato)
Propham *	Herbicide	24	1.94	Arugula, Cauliflower, Celeriac, Celeriac leaves, Cornsalad, Lettuce, Pepper, Potato, Spinach, Tomato	0	0	0.022 ± 0.012	0.010–0.049	0.01 (cornsalad, pepper, potato); 0.02 (celeriac leaves); 0.05 (arugula, cauliflower, celeriac, cucumber, lettuce, pepper, potato, spinach, tomato)
Propiconazole	Fungicide	3	0.24	Celeriac, Pepper, Tomato	0	0	0.017 ± 0.007	0.010–0.026	0.05 (pepper); 0.1 (celeriac); 3 (tomato)
Propoxur	Insecticide	5	0.40	Aubergine, Potato, Tomato	0	0	0.014 ± 0.005	0.010–0.021	0.05 (potato, tomato, aubergine)
Prosulfocarb	Herbicide	3	0.24	Celeriac, Celeriac leaves	0	0	0.030 ± 0.014	0.016–0.049	0.05 (celeriac leaves); 0.08 (celeriac)
Prothioconazole *	Fungicide	37	2.99	Aubergine, Bean, Broccoli, Carrot, Cauliflower, Cucumber, Green bean, Lettuce, Melon	2 (sesame seed, Swiss chard)	5.41	0.015 ± 0.007	0.010–0.040	0.01 (aubergine, cucumber, green bean, lettuce, pepper, spinach, Swiss chard, tomato, watermelon); 0.02 (bean, cauliflower, cucumber, lettuce, melon, onion, pepper, potato, sesame seed, tomato); 0.05 (bean, broccoli, onion); 0.1 (carrot)
Pymetrozine	Insecticide	3	0.24	Bean, Cucumber, Pepper	0	0	0.071 ± 0.044	0.014–0.121	1 (cucumber); 2 (bean); 3 (pepper)
Pyraclostrobin	Fungicide	10	0.81	Beetroot, Broccoli, Cabbage, Lettuce, Pepper, Radish, Tomato	0	0	0.044 ± 0.057	0.010–0.171	0.1 (beetroot, broccoli); 0.2 (cabbage); 0.3 (tomato); 0.5 (pepper, radish); 2 (lettuce)
Pyridaben	Insecticide	4	0.32	Pepper, Tomato	0	0	0.013 ± 0.003	0.010–0.018	0.3 (tomato); 0.5 (pepper)
Pyrimethanil	Fungicide	8	0.65	Cucumber, Tomato	0	0	0.109 ± 0.140	0.032–0.467	0.7 (cucumber); 1 (tomato)
Pyriproxyfen	Insecticide	3	0.24	Pepper, Potato, Tomato	0	0	0.017 ± 0.003	0.014–0.021	0.05 (potato); 1 (pepper, tomato)
Spinetoram A	Insecticide	3	0.24	Arugula, Spinach, Tomato	0	0	0.024 ± 0.008	0.018–0.035	0.05 (arugula); 0.5 (tomato); 1.5 (spinach)
Spinetoram B	Insecticide	2	0.16	Tomato	0	0	–	0.015–0.019	0.5 (tomato)
Spinosad	Insecticide	5	0.40	Arugula, Celeriac leaves, Cucumber, Spinach	0	0	0.208 ± 0.190	0.012–0.498	0.3 (cucumber); 10 (arugula); 15 (celeriac leaves, spinach)
Spinosyn B	Insecticide	4	0.32	Arugula, Lettuce, Spinach	0	0	0.100 ± 0.098	0.010–0.248	10 (arugula, lettuce, spinach)
Spirodiclofen	Insecticide	2	0.16	Pepper, Tomato	0	0	–	0.027–0.028	0.2 (pepper); 0.5 (tomato)
Spiromesifen *	Insecticide	28	2.27	Aubergine, Bean, Carrot, Celeriac, Chinese cabbage, Cornsalad, Cucumber, Ginger, Parsley root, Pepper, Potato, Radish, Tomato	1 (ginger)	3.57	0.019 ± 0.021	0.010–0.128	0.02 (carrot, celeriac, cornsalad, ginger, parsley root, potato, Chinese cabbage, radish); 0.2 (pepper); 0.3 (cucumber); 0.5 (aubergine, pepper, tomato); 1 (tomato, bean)
Spirotetramat	Insecticide	2	0.16	Cucumber, Pepper	0	0	–	0.010–0.019	0.2 (cucumber); 2 (pepper)
Spiroxamine	Fungicide	1	0.08	Tomato	0	0	–	0.012	0.05 (tomato)
Sulfentrazone	Herbicide	1	0.08	Spinach	0	0	–	0.010	0.10 (spinach)
Tebuconazole	Fungicide	8	0.65	Celeriac, Ginger, Pepper, Potato, Tomato	0	0	0.023 ± 0.012	0.010–0.043	0.02 (potato); 0.4 (ginger); 0.5 (celeriac); 0.6 (pepper); 0.9 (tomato)
Tebufenozide	Insecticide	19	1.54	Bean, Cucumber, Ginger, Onion, Potato, Sesame seed, Spinach, Tomato	0	0	0.023 ± 0.027	0.010–0.125	0.05 (bean, cucumber, ginger, onion, potato, sesame seed); 1 (tomato); 10 (spinach)
Tebufenpyrad	Insecticide	5	0.40	Ginger	5 (ginger)	100	0.575 ± 0.254	0.220–0.894	0.05 (ginger)
Teflubenzuron	Insecticide	6	0.49	Bean, Lettuce, Pepper, Tomato	0	0	0.018 ± 0.006	0.013–0.032	0.05 (bean, lettuce); 1 (tomato); 1.5 (pepper)
Tefluthrin	Insecticide	1	0.08	Carrot	0	0	–	0.010	0.05 (carrot)
Terbutryn	Herbicide	3	0.24	Cucumber, Pepper, Potato	0	0	0.010 ± 0.000	0.010	0.01 (cucumber, potato, pepper)
Tetraconazole	Fungicide	5	0.40	Ginger, Lettuce, Tomato	0	0	0.022 ± 0.011	0.010–0.036	0.02 (ginger, lettuce); 0.1 (tomato)
Tetradifon	Insecticide	1	0.08	Ginger	0	0	–	0.010	0.01 (ginger)
Thiabendazole	Fungicide	5	0.40	Cucumber, Potato, Spinach, Tomato	0	0	0.022 ± 0.013	0.012–0.048	0.05 (cucumber, spinach, tomato); 15 (potato)
Thiacloprid *	Insecticide	29	2.35	Arugula, Aubergine, Bean, Brussels sprouts, Cucumber, Garlic, Lettuce, Melon, Pepper, Potato, Spinach, Tomato, Watermelon	0	0	0.083 ± 0.280	0.011–1.548	0.02 (potato, spinach); 0.1 (bean); 0.2 (garlic, melon, watermelon); 0.3 (Brussels sprouts, cucumber); 0.5 (aubergine, cucumber, tomato); 1 (pepper); 2 (lettuce, arugula)
Thiamethoxam	Insecticide	20	1.62	Arugula, Aubergine, Cabbage, Cucumber, Ginger, Lettuce, Onion, Peas, Pepper, Tomato, Watermelon	0	0	0.032 ± 0.044	0.010–0.200	0.01 (arugula, ginger, onion); 0.02 (cabbage); 0.15 (watermelon); 0.2 (aubergine, peas, tomato); 0.5 (cucumber); 0.7 (pepper); 5 (lettuce)
Thiophanate-methyl	Fungicide	2	0.16	Tomato	0	0	–	0.021–0.036	1 (tomato)
Triadimefon	Herbicide	1	0.08	Cucumber	0	0	–	0.010	0.01 (cucumber)
Triadimenol *	Fungicide	8	0.65	Bean, Celeriac, Cucumber, Pepper	1 (cucumber)	12.50	0.020 ± 0.015	0.010–0.055	0.01 (cucumber, pepper); 0.1 (bean); 0.2 (celeriac, cucumber); 1 (pepper)
Trifloxystrobin	Fungicide	1	0.08	Potato	0	0	–	0.011	0.02 (potato)
Triticonazole	Fungicide	1	0.08	Cucumber	0	0	–	0.010	0.01 (cucumber)
Zoxamide	Fungicide	1	0.08	Spinach	1 (spinach)	100	–	0.940	0.02 (spinach)

MRL, maximum residue level according to the regulations of the Republic of Serbia [12,13]. * For these pesticides, the MRL has changed over the years for the same type of vegetable. ** σ—standard deviation.

**Table 3:** Status of pesticide contamination in vegetables reported by different authors across countries.

Name of the Samples	Country (Region or City) of Origin	Year	No. of Samples	No. of Analyzed/ Detected Pesticides	Most Frequently Detected Pesticide Residues	% of Samples with Residues Above the MRL	References
Tomato and eggplant	Sudan (Khartoum)	2017	117	30/16	Permethrin, endosulfan, and carbaryl	18.8% (carbaryl, cypermethrin, endosulfan, imidacloprid, fenvlarate, deltamethrin, diazinon, and dimethoate)	Ali et al. [17]
Tomato	Colombia (Bogota)	2011	400	24	Pyrimethanil, carbendazim, dimetho-morph, and acephate	0.25% (carbendazim)	Arias et al. [18]
Vegetable	Italy (Campania)	October 2010 to April 2011	145	14	Propamocarb, cyprodinil, and boscalid	2.1% (etofenprox, and dimethomorph)	Arienzo et al. [19]
Vegetable and fruit	Turkey (Aegean)		1423 (850 vegetable and 573 fruit)	186/80	Acetamiprid, chlorpyriphos, and carbendazim	9.8%	Bakırcı et al. [20]
Green-leafy vegetable	Turkey (Tokat and Amasya)	January, February, and March 2021	74	260/13	Acetamiprid, pyraclostrobin, cypermethrin, and deltamethrin	6.75% (kresoxim-methyl, metrafenone, pyridaben, and sulfoxaflor)	Balkan and Yılmaz [21]
Vegetable	Nepal (Southern)	Winter period of 2017	86 (27 eggplant, 27 chilli, and 32 tomato)	23/14	Carbendazim, and chloropyrifos	4% (eggplant), 44% (tomato), and 19% (chilli); carbendazim, chloropyrifos, omethoate, and triazophos	Bhandari et al. [22]
Vegetable	Chile and Mexico ()	End of 2018 and beginning of 2019	101	22/11	Lambda-cyhalothrin	13.9% (carbaryl, chlorothalonil, chlorpyrifos, dimethoate, lambda-cyhalothrin, methamidophos, and monocrotophos)	Calderon et al. [23]
Vegetable	Ethiopia (Rift Valley and Akaki Kality—Addis Ababa)	October 2020 to January 2021	232	35/6	Diazinon	15.0% (diazinon, propargite, and profenofos)	Dinede et al. [24]
Ready-to-eat leafy vegetable	Chile (Santiago)	2016–2017	53	6	Carbendazim, chlorpyrifos, cyfluthrin and lambda-cyhalothrin, and methamidophos	11.3% (methamidophos, chlorothalonil, chlorpyrifos, and carbendazim)	Elgueta et al. [25]
Vegetable	Chile (Metropolitana)	August to December 2018	80 (23 tomato and 57 lettuce)	21/20	Methamidophos, methomyl, difenoconazole, cyprodinil, and boscalid	16.3% (acetamiprid, chlorothalonil, difenoconazole, methamidophos, and methomyl)	Elgueta et al. [26]
Leafy vegetable	Chile (North Central)	2014–2015	118	36/19	Methamidophos, chlorpyrifos, boscalid, lambda-cyhalothrin, carbendazim, and imidacloprid	27.1% (carbendazim, chlorpyrifos, difenoconazole, lambda-cyhalothrin, mancozeb, metalaxyl, methamidophos, and thiamethoxam)	Elgueta et al. [27]
Vegetable and fruit	United Arab Emirates	2019	9724 (4343 vegetable and 5381 fruit)	343/93		7.52% (chlorpyrifos, acetamiprid, and phenthoate)	El-Mageed et al. [28]
Vegetable	Egypt	2012	207 (103 pepper and 104 cucumber)	450/64	Paper: chlorpyrifos, profenofos, and carbendazim; Cucumber: metalaxyl, chlorpyrifos, and propamocarb	28.16% (paper: carbendazim, chlorfenapyr, chlorpropham, cyproconazole, diazinon, ethion, famoxadone, flusilazole, methamidophos, methomyl, profenofos, and propargite; 16.35% (cucumber: carbendazim, chlorfenapyr, chlorpyrifos, dicofol, ethion, fenpropathrin, metalaxyl, methomyl, procymidone, propargite, and prothioconazole	Ibrahim et al. [29]
Vegetable	China (Changchun)	August 2017 to April 2018	313	18/15	Organophosphorus	7.99% (carbofuran, dichlorvos, dicofol, fenitrothion, dimethoate, malathion, parathion, permethrin, and phorate)	Jiang et al. [30]
Greenhouse cucumber, cantaloupe, and melon	Iran (Tehran)	May 2020 to March 2021	250 (100 greenhouse cucumber and 150 cantaloupe and melon)	56	Cucumber: thiacloprid, diazinon, tebuconazole, and imidacloprid; Cantaloupe and melon: chlorpyrifos, imidacloprid, thiacloprid, and metalaxyl	20.4% (cucumber: diazinon, imidacloprid, tebuconazole, and thiacloprid; cantaloupe and melon: chlorpyrifos, imidacloprid, metalaxyl, and thiacloprid)	Mahdavi et al. [31]
Wheat grain and its product	Algeria (Algiers, Blida and Boumerdes)		80	11		5.0% (benalaxyl, chlorpyrifos, and metalaxyl)	Mebdoua and Ounane [32]
Vegetable and fruit	China (Mid-western)	2018	253 (123 vegetable and 130 fruit)	11	Carbendazim, difenoconazole, and tebuconazole	0.79% (vegetable: ifenoconazole and procymidone)	Qin et al. [33]
Vegetable		March 2018 to September 2018	211	80/37	Methomyl, imidacloprid, metalaxyl, cyproconazole, carbendazim, triadimenol, profenofos, chlorpyrifos-methyl, malathion, and acetamiprid	20.9% (chlorantraniliprole, chlorfenapyr, cyproconazole, ethion, malathion, metalaxyl, methomyl, myclobutanil, profenofos, and tebuconazole)	Ramadan et al. [34]
Leafy green vegetable	Italy (20 regions)	September 2013 to June 2015	300	210/23	Fungicides	0.67% (paclobutrazol and tau-fluvalinate)	Santarelli et al. [35]
Vegetable	Egypt (Dakahlia)	January to April 2018	176	23	Chlorpyrifos, acetamiprid, and thiamethoxam	16.48%	Shalaby et al. [36]
Vegetable	India (Andaman Islands)	2011	250	15	α-cypermethrin, chlorpyrifos, and endosulfan	5.2% (α-cypermethrin, chlorpyrifos, dimethoate, endosulfan, ethion, monocrotophos, and triazophos)	Swarnam and Velmurugan [37]
Vegetable	China (Shanghai)	2018– 2021	7028	68/36	Dimethomorph, propamocarb, and acetamiprid	0.47% (acetamiprid, bifenthrin, chlorpyrifos, cypermethrin, dimethomorph, imidacloprid, phoxim, procymidone, and thiamethoxam)	Tong et al. [38]
Minor vegetable	China (Guizhou)	January to November 2020	400	97/15	Chlorpyrifos, imidacloprid, acetamiprid, and emamectin benzoate	17.25% (acetamiprid, carbendazim, emamectin benzoate, imidacloprid, pendimethalin, and phoxim)	Wang et al. [39]
Cowpea	China (Hainan)	November 2018 to June 2021	574	35/27	Chlorfenapyr, difenoconazole, cypermethrin, pyridaben, profenofos, chlorpyrifos-ethyl, cyhalothrin, and fenpropathrin	17.1% (chlorfenapyr, cyfluthrin, cyhalothrin, cypermethrin, difenoconazole, fenitrothion, fenpropathrin, fenvalerate, parathion-methyl, and pyridaben)	Zhang et al. [40]
Vegetable	Ghana (Greater Accra)	January and December 2015	155	21/17	Chlorpyrifos, malathion, allethrin, lambda-cyhalothrin, permethrin, and bifenthrin	21% (allethrin, bifenthrin, chlorfenvinphos, chlorpyrifos, cyfluthrin, cypermethrin, deltamethrin, lambdacyhalothrin, malathion, methamidophos, and permethrin)	Blankson et al. [54]
Vegetable and fruit	Egypt (Sharkia)	July 2020 to June 2021	120 (66 vegetable and 54 fruit)	86		35 out of 86 pesticide residues (40.7%) exceeded the MRLs	El-Sheikh et al. [55]
Cowpea	China (Southern)	2013–2014	150	14/7	Acetamiprid, carbofuran, and carbendazim	2.7% (acetamiprid and carbofuran)	Huan et al. [56]
Vegetable	Kenya (Nairobi)	April, May and June 2018	90	118/5	Profenofos, chlorpyrifos, acephate, methamidophos, and omethoate	14.4% (acephate, chlorpyrifos, methamidophos, and omethoate)	Omwenga et al. [57]
Vegetable	United Arab Emirates (Dubai)	2018–2019	5560	262/79	Organophosphorus (acephate, methamidophos, and profenofos), triazole, and pyrethroid pesticide groups	30.5% (acephate, bifenthrin, carbendazim, chlorfenapyr, chlorpyrifos, deltamethrin, dimethoate, hexaconazole, metalaxyl, methamidophos, monocrotophos, omethoate, profenofos, tebuconazole, and triazophos)	Osaili et al. [58]
Leafy vegetable, stalk and stem vegetable	South Korea (Gwangju and Jeonnam)	2010–2014	8496	230/61	Procymidone, dimethomorph, and azoxystrobin	1.4% (31 different pesticides)	Park et al. [59]
Vegetable	China (15 provinces)	July to September 2016	2169	26	Chlorpyrifos, cyhalothrin, and cypermethrin	6.1% (13 different pesticides)	Xu et al. [60]
Vegetable	Korea (Seoul)	2010–2014	34,520	283/105	Azoxystrobin, diethofencarb, procymidone, cypermethrin, and tebufenpyrad	1.4% (45 different pesticides: paclobutrazol, diazinon, chlorpyrifos, endosulfan, and procymidone)	Yi et al. [61]
Vegetable	India (Karnataka)	2011	50	20	Organochlorines, organophosphates, and pyrethroids	58% (organochlorine)	Ananda Gowda and Somashekar [65]
Vegetable	Ghana (Accra)	July 2010 to February 2011	240	9	DDT (o,p’-DDE, o,p’-DDD, and p,p’-DDE), lindane and o,p’-DDT	31.48% (heptachlor + its epoxide, lindane, o,p’-DDE, o,p’-DDD, o,p’-DDT, p,p’-DDE, and p,p’-DDT)	Bempah et al. [66]
Vegetable	Bangladesh (10 regions)	September 2009 to October 2012	210	19	Chlorpyrifos, carbofuran, diazinon, carbaryl, malathion, endosulfan, cypermethrin, and dimethoate	20.0% (carbaryl, carbofuran, chlorpyrifos, diazinon, and endosulfan)	Chowdhury et al. [67]
Nonleafy vegetable	Saudi Arabia (Riyadh)	January 2007 to December 2008	1057	86/43	Permethrin, endosulfan, and deltamethrin	15.89%	EL-Saeid and Selim [68]
Leafy vegetable	Malaysia (Cameron Highlands)		109	15		6.4%	Farina et al. [69]
Vegetable	Vietnam (2 provinces)	May to July 2018	290	24/10	Cypermethrin, difenoconazole, fipronil, and fenobucarb	23.0%	Giang et al. [70]
Vegetable	China (Jilin)	2016–2017	230	18	Organophosphorus	7.39% (organophosphorus)	Hu et al. [71]
Tomato	Iran	2010	80	3		1.25% (dithiocarbamates)	Jafari et al. [72]
Vegetable	Bangladesh	2010–2022	1577		Chlorpyrifos, dimethoate, diazinon, and malathion	21.2%	Khatun et al. [73]
Vegetable	Togo (several regions)		150	20		16.68%	Kolani et al. [74]
Vegetable	Pakistan (Hyderabad)		200	6		61% (carbofuran and chlorpyrifos)	Latif et al. [75]
Vegetable	China (Hebei)	April 2012 to May 2013	226	38/19	Acephate, cyhalothrin, bifenthrin, omethoate, isazophos, dimethoate, chlorpyrifos, and malathion	2.65% (omethoate)	Li et al. [76]
Cucumber and tomato	Kazakhstan (Almaty)	2012–2014	82	184/29	Organochlorine	28.0% (endosulfan, lambda-cyhalothrin, and chlorpyrifos ethyl)	Lozowicka et al. [77]
Brassica vegetable	Poland (north-eastern)	2006–2009	365	130/15	Chlorpyrifos and cypermethrin	9% (chlorpyrifos, boscalid, chlorothalonil, dimethoate, and chlorothalonil)	Łozowicka et al. [78]
Grain	Kazakhstan (Kostanay and Almaty)	2012	80	180	Chlorpyrifos methyl and pirimiphos-methyl	8.75% (10 different pesticides, mainly o,p’-DDE)	Lozowicka et al. [79]
Vegetable	China (Henan)	2020	5576	8/5		1.1%	Ma et al. [80]
Tomato and watermelon	Tanzania (Dar es Salaam)	January to February 2014	24 (12 tomato and 12 watermelon)	18/8		41.7% (tomato); 50% (watermelon)	Mahugija et al. [81]
Vegetable	Saudi Arabia (Al-Qassim)	October 2008 to January 2009	160	23	Carbaryl, biphenyl, and carbofuran	33.13% (carbaryl, carbofuran, propoxur, tolclofos-methyl, paraquat, amitrole, metalaxyl, bromoxynil, chlorpyrifos, dicofol, dieldrin, lindane, and tefluthrin)	Osman et al. [82]
Leafy vegetable	South Korea (Gwangju)	2005–2019	17977	230/19	Azoxystrobin, dimethomorph, and procymidone	2.4% (diniconazole, lufenuron, and procymidone)	Park et al. [83]
Vegetable	China (western)	2010–2013	506	21/17	Cyhalothrin, chlorpyrifos, cypermethrin, omethoate, fenvalerate, bifenthrin, isocarbophos, and parathion-methyl	4.94% (acephate, chlorpyrifos, cypermethrin, isocarbophos, omethoate, and parathion-methyl)	Qin et al. [84]
Tomato	Morocco (Souss Massa Valley)	May 2009 to June 2010	120	8	Endosulfan, deltamethrin, and procymidone	8.3% (endosulfan and deltamethrin)	Salghi [85]
Vegetable	Thailand (Kwan Phayao Lake)	August to September 2013	147	21/10	Chlorpyrifos, diazinon, malathion, and monocrotophos	22.45% (chlorpyrifos, diazinon, monocrotophos, and profenofos)	Sapbamrer and Hongsibsong [86]
Leafy vegetable	Saudi Arabia (Riyadh)	2007–2008	567	86/36		18.34%	Selim et al. [87]
Maize, cowpea and millet	Cameroon (northern)	September to November 2008	82	7/6	Lindane, α-endosulfan, malathion, and β-endosulfan.	˃75% (α-endosulfan, β-endosulfan, lindane, malathion, and permethrin)	Sonchieu et al. [88]
Vegetable	China (Shaanxi)	2010	285	33/25	Omethoate, chlorpyrifos, acephate, dichlorvos, phorate, dimethoate, methidathion, ethoprophos, disulfoton, and tolclofos-methyl	4.56% (chlorpyrifos, ethoprophos, methidathion, omethoate, and phorate)	Wang et al. [89]
Vegetable	China (Changchun)	25 August to 5 September 2014	214	11	Diazinon, phorate, dimethoate, parathion-methyl, omethoate, dichlorvos, fenitrothion, fenthion, parathion, methamidophos, and malathion	23.4% (phorate, parathion, methamidophos, omethoate, dichlorvos, parathion-methyl, diazinon, and fenthion)	Yu et al. [90]
Vegetable	China (northwest)	2011–2013	518	40/32	Malathion, dichlorvos, and dimethoate	7.7% (malathion, dichlorvos, and dimethoate)	Yu et al. [91]

**Table 4:** Number of pesticide residues in an individual sample.

** No. of Pesticide Residues **	**0**	**1**	**2**	**3**	**4**	**5**	**6**	**7**	**8**	**9**	**10**
No. of Samples	740	224	132	52	43	21	15	4	2	2	1
%	59.87	18.12	10.68	4.21	3.48	1.70	1.21	0.32	0.16	0.16	0.08
No. of Samples with Residues Above the MRL	0	4 (4 with 1)	5 (2 with 1; 3 with 2)	4 (4 with 1)	3 (3 with 1)	1 (1 with 1)	2 (1 with 1; 1 with 4)		1 (1 with 1)	1 (1 with 2)	1 (1 with 2)

## Data Availability

The original contributions presented in this study are included in the article/Supplementary Materials. Further inquiries can be directed to the corresponding author.

## Supplementary Materials

The following supporting information can be downloaded at https://www.mdpi.com/article/10.3390/foods15122081/s1, Supplementary Material S1 and Supplementary Material S2, Table S1: Summary of studies dealing with detection of pesticide residue in fresh vegetables/food of plant origin.
