# Supplementary material for: Assessment of Pesticide Residue Content in Fresh Plant-Based Products Available on the Serbian Market Using the QuEChERS Method Combined with LC-MS/MS and GC-MS/MS
Source: Foods. 2026 Jun 8;15(12):2081. doi: 10.3390/foods15122081 (PMC13298267; doi:10.3390/foods15122081)
Supplement: Supplementary file 1 [file foods-15-02081-s001.zip › Supplementary material S2 Table S1.pdf]

Supplementary material S2

**Table S1.** Summary of studies dealing with detection of pesticide residue in fresh vegetables/food of plant origin.

| Reference | Country of origin | Commodity (fresh) | No. of samples | % of samples without detectable residues (< LOQ) | % of samples with residues at or above the LOQ | % of samples with residues above the MRL |
|---|---|---|---|---|---|---|
| Abd-Elhaleem [1] | Saudi Arabia | Tomato and tomato product | 22 | 64 | 36 | 0 (MRLs in EU/Codex) |
| Ahoudi et al. [2] | Togo | Vegetable | 42 | NM | 21.43 (≤ MRL) | 78.57 (MRLs in EU) |
| Al-Antary et al. [3] | Jordan | Vegetable (local samples) | 306 | 92.81 | 7.19 | 0.33 (MRLs in Jordan/Codex) |
| | | Vegetable (imported samples) | 198 | 98.99 | 1.01 | 0 (MRLs in Jordan/Codex) |
| | | Fruit (local samples) | 94 | 82.98 | 17.02 | 2.13 (MRLs in Jordan/Codex) |
| | | Fruit (imported samples) | 202 | 78.71 | 21.29 | 0 (MRLs in Jordan/Codex) |
| Al-Antary et al. [4] | Jordan | Fruit and vegetable | 400 (local samples) | 95.75 | 4.25 | 0.25 (MRLs in Jordan/Codex) |
| | | | 400 (imported samples) | 94.25 | 5.75 | 0.25 (MRLs in Jordan/Codex) |
| Al-Antary et al. [5] | Jordan | Fruit and vegetable | 240 (local samples) | 87.08 | 12.92 | 5.83 (MRLs in Jordan/Codex) |
| | | | 400 (imported samples) | 98.0 | 2.0 | 0.75 (MRLs in Jordan/Codex) |
| Algharibeh and AlFararjeh [6] | Jordan | Fruit and vegetable | 158 | 46 | 54 | 22 (MRLs in EU); some samples contained pesticide residues (propargite) that are banned for use in the Jordan/EU |
| Ali et al. [7] | Sudan | Eggplant | 27 | 3.7 | 96.3 | 18.5 (MRLs in Codex) |

| | | | | | | |
|---|---|---|---|---|---|---|
| | | Tomatoes | 90 | 8.8 | 91.2 | 18.9 (MRLs in Codex) |
| Al-Nasir et al. [8] | Jordan | Citrus fruit and vegetable | 40 | NM | NM | NM (MRLs in EU); in some samples pesticide residues were above MRLs in EU |
| Al-Shamary et al. [9] | Qatar | Fruit and vegetable | 127 | 36.8 | 73.2 | 62.2 (organochlorines pesticides, MRLs in Codex) |
| Ananda Gowda and Somashekar [10] | India | Vegetable | 50 | 0 | 100 | 58 (MRLs in PFA standards) |
| Arias et al. [11] | Colombia | Tomato | 400 | 29.5 | 70.5 | 0.25 (carbendazim, MRLs in Codex) |
| Arienzo et al. [12] | Italy | Vegetable | 145 | 51.7 | 48.3 | 2.1 (MRLs in EU); some samples contained pesticide residues that are unauthorized for use in the EU |
| Badr et al. [13] | Egypt | Fruit and vegetable | 45 | NM | NM | NM (MRLs in EU/Codex/FDA/EPA); in some samples pesticide residues were above MRLs in Egypt |
| Bakırcı et al. [14] | Turkey | Vegetable | 850 | 51 | 49 | 9.8 (MRLs in Turkey) |
| | | Fruit | 573 | 40 | 60 | 8.4 (MRLs in Turkey) |
| Balkan and Yılmaz [15] | Turkey | Leafy vegetables | 74 | 42.4 | 57.6 | 6.75 (MRLs in EU) |
| Bempah et al. [16] | Ghana | Fruit and vegetable | 350 | 37.5 | 62.5 | 19.0 (MRLs in EU) |
| Bempah et al. [17] | Ghana | Vegetable | 240 | 28.10 | 71.90 | 31.48 (MRLs in EU); some samples contained pesticide residues (DDT) that |

| | | | | | | |
|---|---|---|---|---|---|---|
| | | | | | | are banned for use in the Ghana |
| Bhandari et al. [18] | Nepal | Vegetable | 86 | ~ 2 | ~ 97 | 27 (MRLs in EU) |
| Bilaro et al. [19] | Tanzania | Tomato | 42 | 21.49 | 78.51 | 4.67 (cypermethrin, MRL in Codex) |
| Blankson et al. [20] | Ghana | Vegetable | 155 | 48 | 52 | 20 (MRLs in EU) |
| Bojacá et al. [21] | Colombia | Tomato | 26 (open field) | 26.9 | 73.1 | 53.9 (MRLs in EU) |
| | | | 105 (greenhouse) | 28.6 | 71.4 | 27.6 (MRLs in EU) |
| Chen et al. [22] | China | Fruit and vegetable | 3009 | 62.3 | 37.7 | 11.7 (MRLs in China) |
| Choubbane et al. [23] | Morocco | Fruit and vegetable | 51 | 31 | 69 | 0 (MRLs in EU); some samples contained pesticide residues that are banned for use in the Morocco |
| Chowdhury et al. [24] | Bangladesh | Vegetable | 210 | 48.70 | 51.30 | 20.0 (MRLs in EU) |
| Del Prado-Lu [25] | Philippines | Eggplant | 120 | 80 | 20 | NM (MRLs in Codex/EPA/EU) |
| Dinede et al. [26] | Ethiopia | Vegetable | 232 | 52 | 48 | 15 (MRLs in EU) |
| Diop et al. [27] | Senegal | Cabbage | 31 | 7 | 93 | NM (MRLs in EU); in some samples pesticide residues were above MRLs in EU |
| | | Lettuce | 88 | 29 | 71 | NM (MRLs in EU); in some samples pesticide residues were above MRLs in EU |
| | | Tomato | 57 | 35 | 65 | NM (MRLs in EU); in some samples pesticide residues were above MRLs in EU |

| | | | | | | |
|---|---|---|---|---|---|---|
| Duan et al. [28] | China | Cowpea | 433 | NM | NM | NM (MRLs in China); in some samples pesticide residues were above MRLs in China |
| Elgueta et al. [29] | Chile | Ready-to-eat leafy vegetable | 53 | 50.9 | 49.1 | 11.3 (MRLs in Chile) |
| Elgueta et al. [30] | Chile | Tomato | 23 | 48 | 52 | 17.4 (MRLs in Chile) |
| | | Lettuce | 57 | 42 | 58 | 15.8 (MRLs in Chile) |
| Elgueta et al. [31] | Chile | Leafy vegetable | 118 | 65 | 35 | 27.1 (MRLs in Chile); some samples contained pesticide residues that are unauthorized for use in the Chile |
| El-Mageed et al. [32] | United Arab Emirates | Vegetable | 4343 | 65.43 | 34.57 | 7.52 (MRLs in EU/Codex); some samples contained pesticide residues that are banned or unauthorised for use in the United Arab Emirates |
| | | Fruit | 5381 | 61.96 | 38.04 | 5.43 (MRLs in EU/Codex); some samples contained pesticide residues that are banned or unauthorised for use in the United Arab Emirates |
| EL-Saeid and Selim [33] | Saudi Arabia | Nonleafy vegetables | 1057 | NM | 83.90 (≤ MRL) | 15.89 (MRLs in Saudi Arabia) |

| | | | | | | |
|---|---|---|---|---|---|---|
| El-Sheikh et al. [34] | | Tomato and strawberry and their products | 74 | 0 | 100 | NM (MRLs in EU); in some samples pesticide residues were above MRLs in EU |
| El-Sheikh et al. [35] | Egypt | Vegetable | 66 | 33 | 67 | 40.7 (MRLs in EU) |
| | | Fruit | 54 | 39 | 61 | 38.9 (MRLs in EU) |
| Farag et al. [36] | Egypt | Herb, fruit and vegetable | 132 | 45.45 | 54.55 | 0.76 (carbendazim, MRLs in Egypt) |
| Farina et al. [37] | Malaysia | Leafy vegetable | 109 | 14.7 | 85.3 | 6.4 (MRLs in EU) |
| Gaouar et al. [38] | Algeria | Tomato | 30 | 53.3 | 46.7 | 3.3 (MRLs in EU) |
| Ghanbari et al. [39] | Iran | Rice | 30 | 0 | 100 | 100 (MRLs in EU) |
| Giang et al. [40] | Vietnam | Vegetable | 290 | 19.3 | 80.7 | 23.0 (MRLs in Vietnam/Codex) |
| Golge et al. [41] | Turkey | Green pepper and cucumber | 725 | 86.8 | 13.2 | 0 (MRLs in EU) |
| Gondo et al. [42] | Botswana | Fruit and vegetable | 83 | 22 | 78 | 13 (MRLs in EU/Codex); some samples contained pesticide residues that are unauthorised for use in the Botswana |
| Guler et al. [43] | Turkey | Wheat | 36 | NM | NM | NM (MRLs in EU); in some samples pesticide residues (organochlorine pesticides) were above MRLs in EU |
| Hasan et al. [44] | Bangladesh | Vegetable | 100 | 55 | 45 | 0 (MRLs in Codex) |
| | | Fruit | 100 | 60 | 40 | 0 (MRLs in Codex) |
| Hjorth et al. [45] | South America | Fruit and vegetable | 724 | 19 | 80.4 | 8.4 (MRLs in South America/EU) |
| Hu et al. [46] | China | Vegetable | 230 | 80.4 | 19.6 | 7.39 (MRLs in China); some samples contained pesticide residues (organochlorine |

| | | | | | | |
|---|---|---|---|---|---|---|
| | | | | | | pesticides) that are banned for use in the China |
| Huan et al. [47] | China | Cowpea | 150 | 30.0 | 70.0 | 2.7 (MRLs in China); some samples contained pesticide residues that are unauthorised for use in the China |
| Ibrahim et al. [48] | Egypt | Fruit and vegetable | 175 | 20 | 80 | 42 (MRLs in Codex) |
| Ibrahim et al. [49] | Egypt | Pepper | 103 | 19.42 | 80.58 | 28.16 (MRLs in Codex) |
| | | Cucumber | 104 | 27.88 | 72.12 | 16.35 (MRL in Codex) |
| Inonda et al. [50] | Kenya | Kale | 92 | 93.5 | 6.5 | 0 (MRLs in Kenya) |
| | | French bean | 18 | 66.6 | 33.3 | 0 (MRLs in Kenya) |
| Jafari et al. [51] | Iran | Tomato | 80 | 18.8 | 81.2 | 1.25 (dithiocarbamates, MRLs in Codex) |
| Jallow et al. [52] | Kuwait | Fruit and vegetable | 150 | 42 | 58 | 21 (MRLs in Codex); some samples contained pesticide residues (aldrin) that are banned for use in the Kuwait |
| Jardim and Caldas [53] | Brazil | Fruit, vegetable, rice, and bean | 13556 | 51.7 | 48.3 | 2.7 (MRLs in Brazil); some samples contained pesticide residues that are unauthorized for use in the Brazil |
| Jiang et al. [54] | China | Vegetable | 313 | 70.61 | 29.39 | 7.99 (MRLs in China); some samples contained |

| | | | | | | |
|---|---|---|---|---|---|---|
| | | | | | | pesticide residues (organochlorine pesticides) that are banned for use in the China |
| Khatun et al. [55] | Bangladesh | Vegetable | 1577 | < 71.0 | > 29.0 | 21.2 (MRLs in EU) |
| Kim et al. [56] | Korea | Fruit, vegetable, rice and soybean | 232 | 70.3 | 29.7 | 0.9 (MRLs in Korea) |
| Knežević et al. [57] | Croatia | Fruit and vegetable | 866 | 66.2 | 33.8 | 5.3 (MRLs in EU) |
| Kolani et al. [58] | Togo | Vegetable | 150 | 0 | 100 | 16.68 (MRLs in EU);<br>0 (MRLs in Codex); some samples contained pesticide residues that are banned for use in the Togo |
| Latif et al. [59] | Pakistan | Vegetable | 200 | 0 | 100 | 60.5 (MRLs in Pakistan) |
| Le et al. [60] | Vietnam | Mushroom | 210 | 38.57 | 61.43 | NM (MRLs in EU); in some samples pesticide residues were above MRLs in EU |
| Li et al. [61] | China | Vegetable | 226 | 65.93 | 34.07 | 2.65 (MRLs in China); some samples contained pesticide residues (omethoate) that are banned for use in the China |
| Li et al. [62] | China | Fungi | 354 | 14.7 | 85.3 | 0 (MRLs in China/EU) |
| Liang et al. [63] | China | Vegetable | 19966 | 99.2 | 0.8 | 0.2 (fipronil, MRLs in China) |
| | | Fruit | 10551 | 99.8 | 0.2 | 0.05 (fipronil, MRLs in China) |

| | | | | | | |
|---|---|---|---|---|---|---|
| Loha et al. [64] | Ethiopia | Vegetable (tomato and onion) | 40 | NM | NM | NM (MRLs in EU); some samples pesticide residues (organochlorine pesticides) were above MRLs in EU |
| Lozowicka et al. [65] | Kazakhstan | Tomato and cucumber | 82 | 41.5 | 58.5 | 28.0 (MRLs in Kazakhstan); some samples contained pesticide residues (endosulfan) that are banned for use in the Kazakhstan |
| Łozowicka et al. [66] | Poland | Cereal | 89 | 84.2 | 15.8 | 0 (MRLs in EU) |
| Łozowicka et al. [67] | Poland | *Brassica* vegetable | 365 | 67.7 | 32.3 | 9.0 (MRLs in Poland/EU) |
| Lozowicka et al. [68] | Kazakhstan | Grain | 80 | 22.5 | 77.5 | 8.75 (MRLs in EU); some samples contained pesticide residues that are banned for use in the Kazakhstan |
| Lu et al. [69] | USA and China | Fruit and vegetable | 122 | NM | > 57 | NM |
| Luo et al. [70] | China | Fruit and vegetable | 3307 | 69.2 | 30.8 | 1.0 (MRLs in China) |
| Ma et al. [71] | China | Vegetable | 5576 | 89.3 | 10.7 | 1.1 (MRLs in China) |
| Mac Loughlin et al. [72] | Argentina | Fruit and vegetable | 135 | 35 | 65 | 36.3 (MRLs in Argentina); some samples contained pesticide residues (endosulfan) that are banned for use in the Argentina |
| Mahdavi et al. [73] | Iran | Cucumber | 100 | NM | NM | 18 (MRLs in Iran) |
| | | Cantaloupe and melon | 150 | NM | NM | 22 (MRLs in Iran) |
| Mahugija et al. [74] | Tanzania | Tomato and watermelon | 24 | 4.2 | 95.8 | 46 (MRLs in Codex) |

| | | | | | | |
|---|---|---|---|---|---|---|
| Mandal and Singh [75] | India | Cauliflower | 50 | 58 | 42 | 0 (MRLs in India) |
| Matta et al. [76] | Estonia | Fruit, vegetable, cereal, processed product, baby food and products of animal origin | 316 (2008 year) | 47 | 53 | 1.6 (MRLs in EU) |
| | | | 397 (2009 year) | 52 | 48 | 2.3 (MRLs in EU) |
| | | | 286 (2010 year) | 53 | 47 | 2.1 (MRLs in EU) |
| | | | 268 (2011 year) | 65 | 35 | 0.7 (MRLs in EU) |
| Mebdoua and Ounane [77] | Algeria | Wheat grains and its products | 80 | 37.5 | 62.5 | 5.0 (MRLs in Algeria) |
| Mebdoua et al. [78] | Algeria | Fruit and vegetable | 160 | 42.5 | 57.5 | 12.5 (MRLs in EU/Codex) |
| Medina et al. [79] | Argentina | Rice | 100 | 0 | 100 | 36 (epoxiconazole, MRLs in Argentina); 0 (MRLs in EU) |
| Melo et al. [80] | Portugal | Tomato | 20 | 65 | 35 | 0 (MRLs in EU) |
| Meng et al. [81] | China | Vegetable | NM | NM | NM | 0 (MRLs in China) |
| Mert et al. [82] | United Kingdom | Fruit and vegetable | 25822 | 40.2 | 59.8 | 4.0 (MRLs in United Kingdom/EU) |
| Montiel-León et al. [83] | Canada | Fruit and vegetable | 133 | 53 | 47 | 0 (MRLs in Canada/EU) |
| Mtashobya [84] | Tanzania | Vegetable | 36 | NM | NM | 0 (MRLs in Codex); some samples contained pesticide residues (DDT) that are banned for use in the Tanzania |
| Mutengwe et al. [85] | South Africa | Fruit and vegetable | 199 | 68 | 32 | 1 (MRLs in EU); some samples contained pesticide residues that are unregistered for use in the South Africa |

| | | | | | | |
|---|---|---|---|---|---|---|
| Mutengwe et al. [86] | South Africa | Fruit and vegetable | 37838 | 43.54 | 56.46 | 0.32 (MRLs in South African); some samples contained pesticide residues that are unregistered for use in the South Africa |
| Mutengwe et al. [87] | South Africa | Fruit and vegetable | 53 | 67.9 | 32.1 | 1.9 (MRLs in South Africa/EU); some samples contained pesticide residues that are banned or unauthorised for use in the South Africa |
| Mwanja et al. [88] | Zambia | Fruit and vegetable | 30 | 36.7 | 63.3 | 10.0 (MRLs in Codex); some samples contained pesticide residues (dichlorvos) that are banned for use in the Zambia |
| Nasreddine et al. [89] | Lebanon | Foods of plant origin and drinks | 1860 | NM | NM | NM (MRLs in EU); in some samples pesticide residues were above MRLs in EU; some samples contained pesticide residues that are banned for use in the Lebanon |
| Ngabirano and Birungi [90] | Uganda | Vegetable (sprayed) | 28 | NM | NM | 59.52 (MRLs in EU) |
| | | Vegetable (unsprayed) | 4 | NM | NM | 18 (MRLs in EU) |
| | | Vegetable (market samples) | 4 | NM | NM | 8 (MRLs in EU) |
| Omeje et al. [91] | Nigeria | Fruit and vegetable | 60 | NM | NM | NM (MRLs in China); in some |

| | | | | | | |
|---|---|---|---|---|---|---|
| | | | | | | samples pesticide residues were above MRLs in Codex |
| Omwenga et al. [92] | Kenya | Vegetable | 90 | 77.8 | 22.2 | 14.4 (MRLs in EU/Codex) |
| Osaili et al. [93] | United Arab Emirates | Vegetable | 5560 | NM | 69.5 (≤MRL) | 30.5 (MRLs in EU); some samples contained pesticide residues (pyridaben) that are banned for use in the United Arab Emirates |
| Oshatunberu et al. [94] | Nigeria | Grain | 23 | NM | NM | NM (MRLs in EU/Codex); some samples pesticide residues (organochlorine pesticides) were above MRLs in EU/Codex; some samples contained pesticide residues that are banned for use in the Nigeria |
| Osman et al. [95] | Saudi Arabia | Vegetable | 160 | 44.40 | 55.60 | 33.13 (MRLs in Codex) |
| Park et al. [96] | Korea | Vegetable | 1049 | 92.0 | 8.0 | 0.95 (MRLs in Korea) |
| | | Fruit | 97 | 92.8 | 7.2 | 1.03 (MRLs in Korea) |
| Park et al. [97] | South Korea | Leafy vegetable, stalk and stem vegetable | 8496 | 89.5 | 10.5 | 1.4 (MRLs in Korea) |
| Park et al. [98] | South Korea | Leafy vegetable | 17977 | 84.3 | 15.7 | 2.4 (MRLs in South Korea); some samples contained pesticide residues |

| | | | | | | |
|---|---|---|---|---|---|---|
| | | | | | | (endosulfan) that are banned for use in the South Korea |
| Patiño et al. [99] | Colombia | Vegetable | 53 | 51 | 49 | 39 (MRLs in EU); some samples contained pesticide residues that are not approved for use in the Colombia |
| | | Fruit | 47 | 43 | 57 | 44 (MRLs in EU); some samples contained pesticide residues that are not approved for use in the Colombia |
| Poulsen et al. [100] | Denmark | Fruit and vegetable (70%), cereal (15%), samples of animal origin (15%) | 17309 | NM | NM | 2.6 (most frequently in fruit, MRLs in EU) |
| Qin et al. [101] | China | Vegetable | 369 | 70.73 | 29.27 | NM (MRLs in China); in some samples pesticide residues were above MRLs; some samples contained pesticide residues that are banned for use in the China |
| Qin et al. [102] | China | Vegetable | 123 | 60.7 | 39.3 | 1.63 (fungicide, MRLs in China) |
| | | Fruit | 130 | 57.6 | 42.4 | 0 (MRLs in China) |
| Qin et al. [103] | China | Vegetable | 506 | 69.76 | 30.24 | 4.94 (MRLs in China) |
| Ramadan et al. [104] | Saudi Arabia | Vegetable | 211 | 31.3 | 68.7 | 20.9 (MRLs in EU) |
| Salghi et al. [105] | Morocco | Tomato | 120 | NM | NM | 8.3 (MRLs in EU) |
| Santarelli et al. [106] | Italy | Leafy green vegetable | 300 (pre-cut and uncut) | 46.7 | 53.3 | 0.67 (MRLs in EU) |

| | | | | | | |
|---|---|---|---|---|---|---|
| Sapbamrer and Hongsibsong [107] | Thailand | Vegetable from farms | 27 | 29.6 | 70.4 | 59.3 (MRLs in EU); some samples contained pesticide residue (monocrotophos) that are banned for use in the Thailand |
| | | Vegetable from markets | 106 | 74.5 | 25.5 | 13.2 (MRLs in EU) |
| | | Vegetable from supermarket | 14 | 78.6 | 21.4 | 0 (MRLs in EU) |
| Selim et al. [108] | Saudi Arabia | Leafy vegetables | 567 | 75.31 | 24.69 | 18.34 (MRLs in Codex) |
| Shalaby et al. [109] | Egypt | Vegetable | 176 | 36.9 | 63.1 | 16.5 (MRLs in EU) |
| Sharma et al. [110] | India | Cabbage | 413 | 94.4 | 5.6 | 0 (MRLs in India); some samples contained pesticide residue (dicofol) that are banned for use in the India |
| | | Green chilli | 197 | 34.5 | 65.5 | 0 (MRLs in India); some samples contained pesticide residue (dicofol) that are banned for use in the India |
| | | Okra | 356 | 61.0 | 39.0 | 0 (MRLs in India); some samples contained pesticide residue (dicofol) that are banned for use in the India |
| Shin et al. [111] | South Korea | Fruit, vegetable and grain | 115 | 82.6 | 17.4 | 0.87 (dinotefuran, MRLs in South Korea) |
| Shoiful et al. [112] | Indonesia | Vegetables (carrot, cucumber, potato, onion) | 21 | NM | NM | 0 (organochlorine pesticides, MRLs in Codex) |

| | | | | | | |
|---|---|---|---|---|---|---|
| | | Rice | 4 | NM | NM | 0 (organochlorine pesticides, MRLs in Codex) |
| | | Pulses (green bean, soybean) | 8 | NM | NM | 0 (organochlorine pesticides, MRLs in Codex) |
| | | Nut (peanut) | 4 | NM | NM | 0 (organochlorine pesticides, MRLs in Codex) |
| | | Fish (milkfish) | 3 | NM | NM | 0 (organochlorine pesticides, MRLs in Codex) |
| Si et al. [113] | China | Fruit and vegetable | 197 | 5.1 | 94.9 | 0 (MRLs in China) |
| Sinha et al. [114] | India | Vegetable | 50 | NM | NM | NM (MRLs in PFA standards); in some samples pesticide residues were above MRLs in PFA standards |
| Sivaperumal et al. [115] | India | Fruit and vegetable | 286 | NM | 83.6 (< MRL in EU); 96 (< MRL in India) | 16.4 (MRLs in EU); 4.2. (MRL in India) |
| Skovgaard et al. [116] | Bolivia | Lettuce | 10 | 50 | 50 | 20 (MRLs in Codex/EU/USA) |
| | | Onion | 10 | 100 | 0 | 0 (MRLs in Codex/EU/USA) |
| | | Potato | 10 | 100 | 0 | 0 (MRLs in Codex/EU/USA) |
| Skretteberg et al. [117] | Southeast Asia | Fruit and vegetable | 721 | 60 | 40 | 12 (MRLs in EU) |
| Sonchieu et al. [118] | Cameroon | Maize, cowpea and millet | 82 | NM | NM | > 75 (MRLs in Codex); some samples contained pesticide residues (organochlorine |

| | | | | | | |
|---|---|---|---|---|---|---|
| | | | | | | pesticides) that are banned for use in the Cameroon |
| Soydan et al. [119] | Turkey | Fruit and vegetable | 3044 | 72.5 | 27.5 | 11.6 (MRLs in Turkey) |
| Srivastava et al. [120] | India | Vegetable | 20 | NM | NM | NM (MRLs in PFA standards); in some samples pesticide residues were above MRLs in PFA standards |
| Sungur and Tunur [121] | Turkey | Fruit and vegetable | NM | NM | NM | NM (MRLs in Turkey /EU); in some samples pesticide residues were above MRLs in Turkey /EU |
| Swarnam and Velmurugan [122] | India | Vegetable | 250 | 66.0 | 34.0 | 5.2 (MRLs in PFA standards); some samples contained pesticide residues (endosulfan) that are banned for use in the India |
| Szpyrka et al. [123] | Poland | Fruit | 547 | 49.4 | 50.6 | 2.2 (MRLs in Poland/EU); some samples contained pesticide residues that are banned or unauthorised for use in the Poland/EU |
| | | Vegetable | 479 | 79.9 | 20.7 | 1.3 (MRLs in Poland/EU); some samples contained pesticide residues that are banned or unauthorised for |

| | | | | | | |
|---|---|---|---|---|---|---|
| | | | | | | use in the Poland/EU |
| Tao et al. [124] | China | Wheat | 206 | NM | NM | NM (MRLs in China/Codex); in some samples pesticide residues were above MRLs in China/Codex; some samples contained pesticide residues that are banned for use in the China |
| Tong et al. [125] | China | Vegetable | 7028 | 70.79 | 29.21 | 0.47 (MRLs in China); some samples contained pesticide residues (chlorpyrifos) that are prohibited for use in the China |
| Toptanci et al. [126] | Turkey | Fruit and vegetable | 493 | 48.5 | 51.5 | 29.2 (MRLs in Turkey) |
| Tripathy et al. [127] | India | Brinjal | 448 | 63.7 | 36.3 | 0 (MRLs in India) |
| | | Capsicum | 363 | 30.3 | 69.7 | 0 (MRLs in India) |
| | | Tomato | 416 | 69.7 | 30.3 | 0 (MRLs in India) |
| | | Cucurbit | 1093 | 66.0 | 34.0 | 0 (MRLs in India) |
| Walorczyk et al. [128] | Poland | Organic crop (fruit, vegetable…) | 528 | 95.6 | 4.4 | NM (MRLs in Poland/EU); some samples contained pesticide residues that are banned or unauthorised for use in the EU |
| Wang et al. [129] | China | Vegetable | 74029 | 92.3 | 7.70 | 0.48 (carbendazim, over limit ratio, MRLs in China) |

| | | | | | | |
|---|---|---|---|---|---|---|
| | | Fruit | 24607 | 73.6 | 26.4 | 0.20 (carbendazim, over limit ratio, MRLs in China) |
| | | Mushroom | 17275 | 88.7 | 11.3 | 0.03 (carbendazim, over limit ratio, MRLs in China) |
| | | Cereal | 908 | 92.8 | 7.20 | 0.44 (carbendazim, over limit ratio, MRLs in China) |
| | | Tea | 470 | 99.1 | 0.90 | 0 (carbendazim, over limit ratio, MRLs in China) |
| Wang et al. [130] | China | Minor vegetable | 400 | 62 | 38 | 17.25 (MRLs in EU); some samples contained pesticide residues that are banned for use in the China |
| Wang et al. [131] | China | Vegetable | 285 | 93.33 | 6.67 | 4.21 (organophosphorus pesticides, MRLs in China); some samples contained pesticide residues that are not approved for use in the China |
| | | | 285 | 84.56 | 15.44 | 1.40 (pyrethroid pesticide, MRLs in China) |
| Witczak et al. [132] | Poland | Fruit (peel and pulp) | 9 | 0 | 100 | 0 (MRLs in EU) |
| | | Vegetables (peel and pulp) | 10 | 0 | 100 | NM (MRLs in EU); in some samples pesticide residues (organophosphorus pesticides) were above MRLs in EU |

| | | | | | | |
|---|---|---|---|---|---|---|
| Xu et al. [133] | China | Vegetable | 20496 | 91.7 | 8.3 | 0.08 (carbendazim, MRLs in China) 8.1 (carbendazim, MRLs in USA/Japan) |
| Xu et al. [134] | China | Vegetable | 2169 | 58.1 | 41.9 | 6.1 (MRLs in China) |
| Yi et al. [135] | Korea | Vegetable | 34520 | 86.1 | 13.9 | 1.4 (MRLs in Korea); some samples contained pesticide residues (endosulfan) that are banned for use in the Korea |
| Yu et al. [136] | China | Vegetable | 214 | 7.9 | 92.1 | 23.4 (organophosphorus pesticides, MRLs in China); some samples contained pesticide residues that are banned for use in the China |
| Yu et al. [137] | China | Vegetable | 518 | NM | NM | 7.7 (MRLs in China); some samples contained pesticide residues that are banned or unauthorised for use in the China |
| Yuan et al. [138] | China | Vegetable | 2082 | 92.8 | 7.2 | 1.4 (chlorpyrifos, MRLs in China); 0.3% (cypermethrin, MRLs in China) |
| Zhang et al. [139] | China | Cowpea | 574 | 19.2 | 80.8 | 17.1 (MRLs in China); some samples contained pesticide residues |

| | |
|---|---|
| | that are banned for use in the China |

Codex – Codex Alimentarius Commission; LOQ – limit of quantification; MRL – maximum residue level; NM – not mentioned.

**References**


1. Abd-Elhaleem, Z.A. Pesticide residues in tomato and tomato products marketed in Majmaah province, KSA, and their impact on human health. *Environ. Sci. Pollut. Res. Int.* **2020**. *27*, 8526–8534.
2. Ahoudi, H.; Gnandi, K.; Tanouayi, G.; Ouro-Sama, K.; Yorke, J.-C.; Creppy, E.E.; Moesch. C. Assessment of pesticides residues contents in the vegetables cultivated in urban area of Lome (southern Togo) and their risks on public health and the environment, Togo. *Int. J. Biol. Chem. Sci.* **2018**, *12*, 2172–2185.
3. Al-Antary, T.M.; Alawi, M.A.; AlAwamleh, A.M.; Al-Oqlah, K. Pesticides residues in agricultural crops in northern districts of Jordan in 2010/2011. *Fresenius Environ. Bull.* **2018**, *27*, 2427–2431.
4. Al-Antary, T.M.; Alawi, M.A.; Said, M.; Haddad, N. Monitoring of pesticide residues in agricultural crops in southern governorates of Jordan in 2011/2012. *Fresenius Environ. Bull.* **2018**, *27*, 2418–2426.
5. Al-Antary, T.M.; Alawi, M.A.; Shadermah, A.M.; Haddad, N.A. Pesticides residues in agricultural crops in southern governorates of Jordan in 2016 and 2017. *Fresenius Environ. Bull.* **2018**, *27*, 6894–6898.
6. Algharibeh, G.R.; AlFararjeh, M.S. Pesticide residues in fruits and vegetables in Jordan using liquid chromatography/tandem mass spectrometry. *Food Addit. Contam. Part B Surveill.* **2019**, *12*, 65–73.
7. Ali, S.E.A.; Aziz, M.E.A.; Mohamed, S.E. Determination of pesticides residues in eggplant and tomatoes from central marked in Khartoum state using Quechers method and gas liquid chromatography-mass spectrometry. *Biomed. J. Sci. Tech. Res.* 2020, *24*, 18165–18173.
8. Al-Nasir, F.M.; Jiries, A.G.; Al-Rabadi, G.J.; Alu'datt, M.H.; Tranchant, C.C.; Al-Dalain, S.A.; Alrabadi, N.; Madanat, O.Y.; Al-Dmour, R.S. Determination of pesticide residues in selected citrus fruits and vegetables cultivated in the Jordan Valley. *LWT - Food Sci. Technol.* **2020**, *123*, 109005.
9. Al-Shamary, N.M.; Al-Ghouti, M.A.; Al-Shaikh, I.; Al-Meer, S.H.; Ahmad, T.A. Evaluation of pesticide residues of organochlorine in vegetables and fruits in Qatar: statistical analysis. *Environ. Monit. Assess.* **2016**, *188*, 3, 198.
10. Ananda Gowda, S.R.; Somashekar, R.K. Evaluation of pesticide residues in farmgate samples of vegetables in Karnataka, India. *Bull. Environ. Contam. Toxicol.* **2012**, *89*, 626–632.
11. Arias, A.; Bojacáb, C.R.; Ahumada, D.A.; Schrevens, E. Monitoring of pesticide residues in tomato marketed in Bogota, Colombia. *Food Control.* **2014**, *35*, 213–217.
12. Arienzo, M.; Cataldo, D.; Ferrara, L. Pesticide residues in fresh-cut vegetables from integrated pest management by ultra performance liquid chromatography coupled to tandem mass spectrometry. *Food Control.* **2013**, *31*, 108–115.
13. Badr, A.N.; Ahmed, M.B.M.; Amer, M.M.; Thang, V.N.; Fouzy, A.S.M. Pesticides evaluation in Egyptian fruits and vegetables: A safety assessment study. *J. Environ. Sci. Technol.* **2019**, *12*, 81–91.
14. Bakırcı, G.T.; Acay, D.B.Y.; Bakırcı, F.; Ötleş, S. Pesticide residues in fruits and vegetables from the Aegean region, Turkey. *Food Chem.* **2014**, *160*, 379–392.
15. Balkan, T.; Yılmaz, O. Method validation, residue and risk assessment of 260 pesticides in some leafy vegetables using liquid chromatography coupled to tandem mass spectrometry. *Food Chem.* **2022**, *384*, 132516.
16. Bempah, C.K.; Buah-Kwofie, A.; Denutsui, D.; Asomaning, J.; Tutu, A.O. Monitoring of pesticide residues in fruits and vegetables and related health risk assessment in Kumasi metropolis, Ghana. *Res. J. Environ. Earth Sci.* **2011**, *3*, 761–771.

17. Bempah, C.K.; Buah-Kwofie, A.; Enimi, E.; Blewu, B.; Agyei-Martey, G. Residues of organochlorine pesticides in vegetables marketed in Greater Accra Region of Ghana. *Food Control.* **2012**, *25*, 537–542.
18. Bhandari, G.; Zomer, P.; Atreya, K.; Mol, H.G.J.; Yang, X.; Geissen, V. Pesticide residues in Nepalese vegetables and potential health risks. *Environ. Res.* **2019**, *172*, 511–521.
19. Bilaro, J.S.; Materu, S.F.; Temba, B.A. Dietary risk assessment of selected organophosphorus and pyrethoid pesticide residues in fresh harvested tomatoes at Makambako Town, Njombe region, Tanzania. *Food Addit. Contam. Part B Surveill.* **2022**, *15*, 235–243.
20. Blankson, G.K.; Osei-Fosu, P.; Adeendze, E.A.; Ashie, D. Contamination levels of organophosphorus and synthetic pyrethroid pesticides in vegetables marketed in Accra, Ghana. *Food Control.* **2016**, *68*, 174–180.
21. Bojacá, C.R.; Arias, L.A.; Ahumada, D.A.; Casilimas, H.A.; Schrevens, E. Evaluation of pesticide residues in open field and greenhouse tomatoes from Colombia. *Food Control.* **2013**, *30*, 400–403.
22. Chen, C.; Qian, Y.; Chen, Q.; Tao, C.; Li, C.; Li, Y. Evaluation of pesticide residues in fruits and vegetables from Xiamen, China. *Food Control.* **2011**, *22*, 1114–1120.
23. Choubbane, H.; Ouakhssase, A.; Chahid, A.; Taourirte, M.; Aamouche, A. Pesticides in fruits and vegetables from the Souss Massa region, Morocco. *Food Addit. Contam. Part B Surveill.* **2022**, *15*, 79–88.
24. Chowdhury, M.A.Z.; Fakhruddin, A.N.M.; Islam, Md.N.; Moniruzzaman, M.; Gan, S.H.; Alam, Md.K. Detection of the residues of nineteen pesticides in fresh vegetable samples using gas chromatography–mass spectrometry. *Food Control.* **2013**, *34*, 457–465.
25. Del Prado-Lu, J.L. Insecticide residues in soil, water, and eggplant fruits and farmers' health effects due to exposure to pesticides. *Environ. Health Prev. Med.* **2015**, *20*, 53–62.
26. Dinede, G.; Bihon, W.; Gazu, L.; Mbokou, S.F.; Girma, S.; Srinivasan, R.; Roothaert, R.; Grace, D.; Gashaw, H.; Knight-Jones, T.J.D. Assessment of pesticide residues in vegetables produced in central and eastern Ethiopia. *Front. Sustain. Food Syst.* **2023**, *7*, 1143753.
27. Diop, A.; Diop, Y.M.; Thiar, D.D.; Cazier, F.; Sarr, S.O.; Kasprowiak, A.; Landy, D.; Delattre, F. Monitoring survey of the use patterns and pesticide residues on vegetables in the Niayes zone, Senegal. *Chemosphere.* **2016**, *144*, 1715–1721.
28. Duan, Y.; Guan, N.; Li, P.; Li, J.; Luo, J. Monitoring and dietary exposure assessment of pesticide residues in cowpea (*Vigna unguiculata* L. Walp) in Hainan, China. *Food Control.* **2016**, 59, 250–255.
29. Elgueta, S.; Fuentes, M.; Valenzuela, M.; Zhao, G.; Liu, S.; Lu, H.; Correa, A. Pesticide residues in ready-to-eat leafy vegetables from markets of Santiago, Chile, and consumer's risk. *Food Addit. Contam. Part B Surveill.* **2019**, *12*, 259–267.
30. Elgueta, S.; Valenzuela, M.; Fuentes, M.; Meza, P.; Manzur, J.P.; Liu, S.; Zhao, G.; Correa, A. Pesticide residues and health risk assessment in tomatoes and lettuces from farms of Metropolitan Region Chile. *Molecules.* **2020**, *25*, 355.
31. Elgueta, S.; Moyano, S.; Sepúlveda, P.; Quiroz, C.; Correa, A. Pesticide residues in leafy vegetables and human health risk assessment in North Central agricultural areas of Chile. *Food Addit. Contam. Part B Surveill.* **2017**, *10*, 105–112.
32. El-Mageed, N.M.A.; Abu-Abdoun, I.I.; Janaan, A.S. Monitoring of pesticide residues in imported fruits and vegetables in United Arab Emirates during 2019 (2020). *Int. Res. J. Pure Appl. Chem.* **2020**, *21*, 239–260.
33. EL-Saeid, M.H.; Selim, M.T. Multiresidue analysis of 86 pesticides using gas chromatography mass spectrometry: II-nonleafy vegetables. *J. Chem.* **2013**, *3*, 727149.
34. El-Sheikh, El-S.A.; Li, D.; Hamed, I.; Ashour, M.-B.; Hammock, B.D. Residue analysis and risk exposure assessment of multiple pesticides in tomato and strawberry and their products from markets. *Foods*. **2023**, *12*, 1936.
35. El-Sheikh, El-S.A.; Ramadan, M.M.; El-Sobki, A.E.; Shalaby, A.A.; McCoy, M.R.; Hamed, I.A.; Ashour, M.-B.; Hammock, B.D. Pesticide residues in vegetables and fruits from farmer markets and associated dietary risks. *Molecules.* **2022**, *27*, 8072.

36. Farag, R.S.; Abdel Latif, M.S.; Abd El-Gawad, A.E.; Dogheim, S.M. Monitoring of pesticide residues in some Egyptian herbs, fruits and vegetables. *Int. Food Res. J.* **2011**, *18*, 659–665.
37. Farina, Y.; Abdullah, Md.P.; Bibi, N.; Khalik, W.M.A.W.M. Determination of pesticide residues in leafy vegetables at parts per billion levels by a chemometric study using GC–ECD in Cameron Highlands, Malaysia. *Food Chem.* **2017**, *224*, 55–61.
38. Gaouar, Z.L.; Chefirat, B.; Saadi, R.; Djelad, S.; Rezk-Kallah, H. Pesticide residues in tomato crops in Western Algeria. *Food Addit. Contam. Part B Surveill.* **2021**, *14*, 281–286.
39. Ghanbari, F.; Moattar, F.; Monavari, S.M.; Arjmandi, R. Human health risk assessment of organophosphorus pesticide in rice crop from selected districts of Anzali International Wetland basin, Iran. *Hum. Exp. Toxicol.* **2016**, 36, 438–444.
40. Giang, C.N.D.; Le, D.B.C.; Nguyen, V.H.; Hoang, T.L.; Tran, T.V.T.; Huynh, T.P.L.; Nguyen, T.Q.T. Assessment of pesticide use and pesticide residues in vegetables from two provinces in Central Vietnam. *PLoS ONE.* **2022**, *17*, e0269789.
41. Golge, O.; Hepsag, F.; Kabak, B. Health risk assessment of selected pesticide residues in green pepper and cucumber. *Food Chem. Toxicol.* **2018**, *121*, 51–64.
42. Gondo, T.F.; Kamakama, M.; Oatametse, B.; Samu, T.; Bogopa, J.; Keikotlhaile, B.M. Pesticide residues in fruits and vegetables from the southern part of Botswana. *Food Addit. Contam. Part B Surveill.* **2021**, *14*, 271–280.
43. Guler, G.O.; Cakmak, Y.S.; Dagli, Z.; Aktumsek, A.; Ozparlak, H. Organochlorine pesticide residues in wheat from Konya region, Turkey. *Food Chem. Toxicol.* **2010**, *48*, 1218–1221.
44. Hasan, G.M.M.A.; Das, A.K.; Satter, M.A. Human health risk assessment through the detection of organochlorine pesticides in vegetables and fruits from Dhaka, Bangladesh by gas chromatography tandem mass spectrometry (GC-MS/MS). *Curr. Res. Nutr. Food Sci.* **2022**, *10*, 720–732.
45. Hjorth, K.; Johansen, K.; Holen, B.; Andersson, A.; Christensen, H.B.; Siivinen, K.; Toome, M. Pesticide residues in fruits and vegetables from South America – A Nordic project. *Food Control.* **2011**, *22*, 1701-1706.
46. Hu, D.; Jiang, M.; Ge, T.; Liu, X.; Li, Z.; Liu, J.; Zhu, K. Pesticide residues in vegetables in four regions of Jilin Province. *Int. J. Food Prop.* **2020**, *23*, 1150–1157.
47. Huan, Z.; Xu, Z.; Luo, J.; Xie, D. Monitoring and exposure assessment of pesticide residues in cowpea (*Vigna unguiculata* L. Walp) from five provinces of southern China. *Regul. Toxicol. Pharmacol.* **2016**, *81*, 260–267.
48. Ibrahim, M.A.; Belal, M.H.; Abdallah, I.S.; El-Sawi, S.A.M. Monitoring and risk assessment of pesticide residues in some locally produced vegetables and fruits. *Egypt. J. Chem.* **2022**, *65*, 429–439.
49. Ibrahim, N.M.; Eweis, E.A.; El-Sawi, S.A.M.; Nassar, K.R.A. Monitoring and risk assessment of pesticide residues in some vegetables in Egypt. *Middle East J. Appl. Sci.* **2018**, *8*, 669–679.
50. Inonda, R.; Njage, E.; Ngeranwa, J.; Mutai, C. Determination of pesticide residues in locally consumed vegetables in Kenya. *Afr. J. Pharmacol. Ther.* **2015**, *4*, 1–6.
51. Jafari, A.; Shoeibi, Sh.; Amini, M.; Amirahmadi, M.; Rastegar, H.; Ghaffarian, A.; Ghazi-Khansari, M. Monitoring dithiocarbamate fungicide residues in greenhouse and non-greenhouse tomatoes in Iran by HPLC-UV. *Food Addit. Contam. Part B Surveill.* **2012**, *5*, 87–92.
52. Jallow, M.F.A.; Awadh, D.G.; Albaho, M.S.; Devi, V.Y.; Ahmad, N. Monitoring of pesticide residues in commonly used fruits and vegetables in Kuwait. *Int. J. Environ. Res. Public Health.* **2017**, *14*, 833.
53. Jardim, A.N.O.; Caldas, E.D. Brazilian monitoring programs for pesticide residues in food – Results from 2001 to 2010. *Food Control.* **2012**, *25*, 607–616.
54. Jiang, M.; Gao, H.; Liu, X.; Wang, Y.; Lan, J.; Li, Y.; Lv, S.; Zhu, K.; Gong, P. Detection of pesticide residues in vegetables sold in Changchun city, China. *J. Food Prot.* **2021**, *84*, 481–489.

55. Khatun, P.; Islam, A.; Sachi, S.; Islam, Md.Z.; Islam, P. Pesticides in vegetable production in Bangladesh: A systemic review of contamination levels and associated health risks in the last decade. *Toxicol. Rep.* **2023**, *11*, 199–211.
56. Kim, J.-Y.; Lee, S.-M.; Lee, H.-J.; Chang, M.-I.; Kang, N.-S.; Kim, N.-S.; Kim, H.; Cho, Y.-J.; Jeong, J.; Kim, M.K.; Rhee, G.-S. Monitoring and risk assessment of pesticide residues for circulated agricultural commodities in Korea-2013. *J. Appl. Biol. Chem.* **2014**, *57*, 235–242.
57. Knežević, Z.; Serdar, M.; Ahel, M. Risk assessment of the intake of pesticides in Croatian diet. *Food Control.* **2012**, *23*, 59–65.
58. Kolani, L.; Mawussi, G.; Sanda, K. Assessment of organochlorine pesticide residues in vegetable samples from some agricultural areas in Togo. *Am. J. Anal. Chem.* **2016**, *7*, 332–341.
59. Latif, Y.; Sherazi, S.T.H.; Bhanger, M.I. Assessment of pesticide residues in commonly used vegetables in Hyderabad, Pakistan. *Ecotoxicol. Environ. Saf.* **2011**, *74*, 2299–2303.
60. Le, L.H.T.; Tran-Lam, T.-T.; Cam, T.Q.; Nguyen, T.N.; Dao, Y.H. Pesticides in edible mushrooms in Vietnam. *Food Addit. Contam. Part B Surveill.* **2021**, *14*, 139–148.
61. Li, W.; Tai, L.; Liu, J.; Gai, Z.; Ding, G. Monitoring of pesticide residues levels in fresh vegetable form Heibei Province, North China. *Environ. Monit. Assess.* **2014**, *186*, 6341–6349.
62. Li, Y., Qin, G.; He, F.; Zou, K.; Zuo, B.; Liu, R.; Zhang, W.; Yang, B.; Zhao, G.; Jia, G. Investigation and analysis of pesticide residues in edible fungi produced in the mid-western region of China. *Food Control.* **2022**, *136*, 108857.
63. Liang, S.-x.; Zhao, Z.; Fan, C.-l.; Xu, J.-z.; Li, H.; Chang, Q.-y.; Pang, G.-f. Fipronil residues and risk assessment of Chinese marketed fruits and vegetables: A long-term investigation over 6 years. *Food Control.* **2019**, *106*, 106734.
64. Loha, K.M.; Lamoree, M.; de Boer, J. Pesticide residue levels in vegetables and surface waters at the Central Rift Valley (CRV) of Ethiopia. *Environ. Monit. Assess.* **2020**, *192*, 546.
65. Lozowicka, B.; Abzeitova, E.; Sagitov, A.; Kaczynski, P.; Toleubayev, K.; Li, A. Studies of pesticide residues in tomatoes and cucumbers from Kazakhstan and the associated health risks. *Environ. Monit. Assess.* **2015**, *187*, 609.
66. Łozowicka, B., Miciński, J.; Zwierzchowski, G.; Kowalski, I.M.; Szarek, J. Monitoring study of pesticide residues in cereals and foodstuff from Poland. *Pol. J. Environ. Stud.* **2012**, *21*, 1703–1712.
67. Łozowicka, B.; Jankowska, M.; Kaczyński, P. Pesticide residues in *Brassica* vegetables and exposure assessment of consumers. *Food Control.* **2012**, *25*, 561–575.
68. Lozowicka, B.; Kaczynski, P.; Paritova, A.E.; Kuzembekova, G.B.; Abzhalieva, A.B.; Sarsembayeva, N.B.; Alihan, K. Pesticide residues in grain from Kazakhstan and potential health risks associated with exposure to detected pesticides. *Food Chem. Toxicol.* **2014**, *64*, 238–248.
69. Lu, C.; Chang, C.-H.; Palmer, C.; Zhao, M.; Zhang, Q. Neonicotinoid residues in fruits and vegetables: An integrated dietary exposure assessment approach. *Environ. Sci. Technol.* **2018**, *52*, 3175–3184.
70. Luo, X.; Zeng, X.; Wei, D.; Ma, C.; Li, J.; Guo, X.; Cheng, L.; Mao, Z. Pesticide residues in common fruits and vegetables in Henan Province, China. *Food Addit. Contam. Part B Surveill.* **2023**, *16*, 244–252.
71. Ma, C.; Wei, D.; Liu, P.; Fan, K.; Nie, L.; Song, Y.; Wang, M.; Wang, L.; Xu, Q.; Wang, J.; Shi, J.; Geng, J.; Zhao, M.; Jia, Z.; Huan, C.; Huo, W.; Wang, C.; Mao, Z.; Huang, S.; Zeng, X. Pesticide residues in commonly consumed vegetables in Henan Province of China in 2020. *Front Public Health. 2022*, *10*, 901485.
72. Mac Loughlin, T.M.; Peluso, Ma.L; Etchegoyen, Ma.A.; Alonso, L.L.; de Castro, Ma.C.; Percudani, Ma.C.; Marino, D.J.G. Pesticide residues in fruits and vegetables of the argentine domestic market: Occurrence and quality. *Food Control.* **2018**, *93*, 129–138.
73. Mahdavi, V.; Eslami, Z.; Gordan, H.; Ramezani, S.; Peivasteh-roudsari, L.; Ma′mani, L.; Khaneghah, A.M. Pesticide residues in green-house cucumber, cantaloupe, and melon samples from Iran: A risk assessment by Monte Carlo Simulation. *Environ. Res.* **2022**, *206*, 112563.

74. Mahugija, J.A.M.; Khamis, F.A.; Lugwisha, E.H.J. Assessment of pesticide residues in tomatoes and watermelons (fruits) from markets in Dar es Salaam, Tanzania. *J. Appl. Sci. Environ. Manage.* **2017**, *21*, 497–501.
75. Mandal, K.; Singh, B. Magnitude and frequency of pesticide residues in farmgate samples of cauliflower in Punjab, India. *Bull. Environ. Contam. Toxicol.* **2010**, *85*, 423–426.
76. Matta, D.; Pehme, S.; Peetsmann, E.; Luik, A.; Meremäe, K. Pesticide residues in Estonian local and imported food in 2008–2011. *Acta Agric. Scand. - B Soil Plant Sci.* **2013**, *63*, Suppl. 1, 78–84.
77. Mebdoua, S.; Ounane, G. Evaluation of pesticide residues in wheat grains and its products from Algeria. *Food Addit. Contam. Part B Surveill.* **2019**, *12*, 1–7.
78. Mebdoua, S.; Lazali, M.; Ounane, S.M.; Tellah, S.; Nabi, F.; Ounane, G. Evaluation of pesticide residues in fruits and vegetables from Algeria. *Food Addit. Contam. Part B Surveill.* **2017**, *10*, 91–98.
79. Medina, M.B.; Munitza, M.S.; Resnik, S.L. Pesticides in randomly collected rice commercialised in Entre Ríos, Argentina. *Food Addit. Contam. Part B Surveill.* **2019**, *12*, 252–258.
80. Melo, A.; Cunha, S.C.; Mansilha, C.; Aguiar, A.; Pinho, O.; Ferreira, I.M.P.L.V.O. Monitoring pesticide residues in greenhouse tomato by combining acetonitrile-based extraction with dispersive liquid–liquid microextraction followed by gas-chromatography–mass spectrometry. *Food Chem.* **2012**, *135*, 1071–1077.
81. Meng, X.; Wang, L.; Wang, N.; Chen, L.; Huang, Q. Investigation and analysis of pesticide residues in four common vegetables and risk assessment of dietary exposure in Ceramic Capital, China. *Molecules.* **2022**, *27*, 6562.
82. Mert, A.: Qi, A.; Bygrave, A.; Stotz, H.U. Trends of pesticide residues in foods imported to the United Kingdom from 2000 to 2020. *Food Control.* **2022**, *133*, 108616.
83. Montiel-León, J.M.; Duy, S.V.; Munoz, G.; Verner, M.-A.; Hendawi, M.Y., Moya, H.; Amyot, M.; Sauvé, S. Occurrence of pesticides in fruits and vegetables from organic and conventional agriculture by QuEChERS extraction liquid chromatography tandem mass spectrometry. *Food Control.* **2019**, *104*, 74–82.
84. Mtashobya, L.A. Assessment of pesticide residues in vegetables from the Western Usambara and Uruguru Mountains in Tanzania. *Environ. Monit. Assess.* **2017**, *189*, 519.
85. Mutengwe, M.T.; Chidamba, L.; Korsten, L. Monitoring pesticide residues in fruits and vegetables at two of the biggest fresh produce markets in Africa. *J. Food Prot.* **2016**, *79*, 1938–1945.
86. Mutengwe, M.T.; Chidamba, L.; Korsten, L. Pesticide residue monitoring on South African fresh produce exported over a 6-year period. *J. Food Prot.* **2016**, *79*, 1759–1766.
87. Mutengwe, M.T.; Aneck-Hahn, N.H.; Korsten, L.; van Zijl, M.C.; de Jager, C. Pesticide residues and estrogenic activity in fruit and vegetables sampled from major fresh produce markets in South Africa. *Food Addit. Contam. Part A Chem. Anal. Control Expo. Risk Assess.* **2016**, *33*, 95–104.
88. Mwanja, M.; Jacobs, C.; Mbewe, A.R.; Munyinda, N.S. Assessment of pesticide residue levels among locally produced fruits and vegetables in Monze district, Zambia. Int. J. Food Contam. **2017**, *4*, 11.
89. Nasreddine, L.; Rehaime, M.; Kassaify, Z.; Rechmany, R.; Jaber, F. Dietary exposure to pesticide residues from foods of plant origin and drinks in Lebanon. *Environ. Monit. Assess.* **2016**, *188*, 485.
90. Ngabirano, H.; Birungi, G. Pesticide residues in vegetables produced in rural south-western Uganda. *Food Chem.* **2022**, *370*, 130972.
91. Omeje, J.S.; Asegbeloyin, J.N.; Ihedioha, J.N.; Ekere, N.R.; Ochonogor, A.E.; Abugu, H.O.; Alum, O.L. Monitoring of pesticide residues in fresh fruits and vegetables available in Nigerian markets and assessment of their associated health risks. *Environ. Monit. Assess.* **2022**, *194*, 516.

92. Omwenga, I.; Kanjab, L.; Zomerd, P.; Louissed, J.; Rietjens, I.M.C.M.; Mold, H. Organophosphate and carbamate pesticide residues and accompanying risks in commonly consumed vegetables in Kenya. *Food Addit. Contam. Part B Surveill.* **2021**, *14*, 48–58.
93. Osaili, T.M.; Al Sallagi, M.S.; Dhanasekaran, D.K.; Bani Odeh, W.A.M.; Al Ali, H.J.; Al Ali, A.A.S.A.; Ismail, L.C.; Al. Mehri, K.O.; Pisharath, V.A.; Holley, R.; Obaid, R.S. Pesticide residues in fresh fruits imported into the United Arab Emirates. *Heliyon.* **2022**, 8, e11946.
94. Oshatunberu, M.A.; Oladimeji, A.; Henry, S.O.; Olaniyan, O.A.; Raimi, M.O. Concentrations of pesticides residues in grain sold at selected markets of Southwest Nigeria. *Nat. Resour. Human Health.* **2023**, *3*, 387–402.
95. Osman, K.A.; Al-Humaid, A.M.; Al-Rehiayani, S.M.; Al-Redhaiman, K.N. Monitoring of pesticide residues in vegetables marketed in Al-Qassim region, Saudi Arabia. *Ecotoxicol. Environ. Saf.* **2010**, *73*, 1433–1439.
96. Park, B.K.; Kwon, S.H.; Yeom, M.S.; Joo, K.S.; Heo, M.J. Detection of pesticide residues and risk assessment from the local fruits and vegetables in Incheon, Korea. *Sci. Rep.* **2022**, *12*, 9613.
97. Park, D.W.; Kim, K.G.; Choi, E.A.; Kang, G.R.; Kim, T.S.; Yang, Y.S.; Moon, S.J.; Ha, D.R.; Kim, E.S.; Cho, B.S. Pesticide residues in leafy vegetables, stalk and stem vegetables from South Korea: a long-term study on safety and health risk assessment. *Food Addit. Contam. Part A Chem. Anal. Control Expo. Risk Assess.* **2016**, *33*, 105–118.
98. Park, D.W.; Yang, Y.S.; Lee, Y.-U.; Han, S.J.; Kim, H.J.; Kim, S.-H.; Kim, J.P.; Cho, S.J.; Lee, D.; Song, N.; Han, Y.; Kim, H.H.; Cho, B.-S.; Chung, J.K.; Kim, A.G. Pesticide residues and risk assessment from monitoring programs in the largest production area of leafy vegetables in South Korea: A 15-year study. *Foods.* **2021**, *10*, 425.
99. Patiño, M.; Valencia-Guerrero, M.F.; Barbosa-Ángel, E.S.; Martínez-Cordón, M.J.; Donado-Godoy, P. Evaluation of chemical and microbiological contaminants in fresh fruits and vegetables from peasant markets in Cundinamarca, Colombia. *J. Food Prot.* **2020**, *83*, 1726–1737.
100. Poulsen, M.E.; Andersen, J.H.; Petersen, A.; Jensen, B.H. Results from the Danish monitoring programme for pesticide residues from the period 2004–2011. *Food Control.* **2017**, *74*, 25–33.
101. Qin, G.; Zou, K.; Li, Y.; Chen, Y.; He, F.; Ding, G. Pesticide residue determination in vegetables from western China applying gas chromatography with mass spectrometry. *Biomed. Chromatogr.* **2016**, *30*, 1430–1440.
102. Qin, G.; Chen, Y.; He, F.; Yang, B.; Zou, K.; Shen, N.; Zuo, B.; Liu, R.; Zhang, W.; Li, Y. Risk assessment of fungicide pesticide residues in vegetables and fruits in the mid-western region of China. *J. Food Compos. Anal.* **2021**, *95*, 103663.
103. Qin, G.; Li, Y.; Chen, Y.; Sun, Q.; Zuo, B.; He, F.; Shen, N.; Jia, G.; Ding, G. Pesticide residues determination in China vegetables in 2010–2013 applying gas chromatography with mass spectrometry. *Food. Res. Int.* **2015**, *72*, 161–167.
104. Ramadan, M.F.A.; Abdel-Hamid, M.M.A.; Altorgoman, M.M.F.; AlGaramah, H.A.; Alawi, M.A.; Shati, A.A.; Shweeta, H.A.; Awwad, N.S. Evaluation of pesticide residues in vegetables from the Asir Region, Saudi Arabia. *Molecules.* **2020**, *25*, 205.
105. Salghi, R.; Luis, G.; Rubio, C.; Hormatallah, A.; Bazzi, L.; Gutiérrez, A.J.; Hardisson, A. Pesticide residues in tomatoes from greenhouses in Souss Massa Valley, Morocco. *Bull. Environ. Contam. Toxicol.* **2012**, *88*, 358–361.
106. Santarelli, G.A.; Migliorati, G.; Pomilio, F.; Marfoglia, C.; Centorame, P.; D'Agostino, A.; D'Aurelio, R.; Scarpone, R.; Battistelli, N.; Di Simone, F.; Aprea, G.; Iannetti, L. Assessment of pesticide residues and microbial contamination in raw leafy green vegetables marketed in Italy. Food Control. **2018**, *85*, 350–335.
107. Sapbamrer, R.; Hongsibsong, S. Organophosphorus pesticide residues in vegetables from farms, markets, and a supermarket around Kwan Phayao Lake of Northern Thailand. *Arch. Environ. Contam. Toxicol.* **2014**, *67*, 60–67.
108. Selim, M.T.; EL-Saeid, M.; Al-Dossari, I.M. Multi-residues analysis of pesticides using gas chromatography mass spectrometry: I- Leafy vegetables. *Res. J. Environ. Sci.* **2011**, *5*, 248–258.
109. Shalaby, S.E.M.; Abdou, G.Y.; El-Metwally, I.M.; Abou-elella, G.M.A. Health risk assessment of pesticide residues in vegetables collected from Dakahlia, Egypt. *J. Plant Prot. Res.* **2021**, 61, 254–264.

110. Sharma, K.K.; Tripathy, V.; Sharma, K.; Gupta, R.; Yadav, R.; Devi, S.; Walia, S. Long–term monitoring of 155 multi–class pesticide residues in Indian vegetables and their risk assessment for consumer safety. *Food Chem.* **2022**, *373*, 131518.
111. Shin, J.m.; Choi, S.-J.; Park, Y.h.; Kwak, B.; Moon, S.H.; Yoon, Y.T.; Jo, S.A.; Yi, H.; Kim, S.j.; Park, S.K.; Park, J.s. Comparison of QuEChERS and Liquid–Liquid extraction methods for the simultaneous analysis of pesticide residues using LC-MS/MS. *Food Control.* **2022**, *141*, 109202.
112. Shoiful, A.; Fujita, H.; Watanabe, I.; Honda, K. Concentrations of organochlorine pesticides (OCPs) residues in foodstuffs collected from traditional markets in Indonesia. *Chemosphere.* **2013**, *90*, 1742–1750.
113. Si, W.-S.; Wang, S.-Y.; Zhang, Y.-D.; Kong, C.; Bai, B. Pesticides and risk assessment in Shanghai fruit and raw eaten vegetables. *Food Addit. Contam. Part B Surveill.* **2021**, *14*, 245–255.
114. Sinha, S.N.; Rao, M.V.V.; Vasudev, K. Distribution of pesticides in different commonly used vegetables from Hyderabad, India. *Food. Res. Int.* **2012**, *45*, 161–169.
115. Sivaperumal, P.; Anand, P.; Riddhi, L. Rapid determination of pesticide residues in fruits and vegetables, using ultra-high-performance liquid chromatography/time-of-flight mass spectrometry. *Food Chem.* **2015**, *168*, 356–365.
116. Skovgaard, M.; Encinas, S.R.; Jensen, O.C.; Andersen, J.H.; Condarco, G.; Jørs, E. Pesticide residues in commercial lettuce, onion, and potato samples from Bolivia–A threat to public health? *Environ. Health Insights.* **2017**, *11*, 1–8.
117. Skretteberg, L.G.; Lyrån, B.; Holen, B.; Jansson, A.; Fohgelberg, P.; Siivinen, K.; Andersen, J.H.; Jensen, B.H. Pesticide residues in food of plant origin from Southeast Asia – A Nordic project. *Food Control.* **2015**, *51*, 225–235.
118. Sonchieu, J.; Ngassoum, M.B.; Tchatchueng, J.B.; Srivastava, A.K.; Srivastava, L.P. Survey of pesticide residues in maize, cowpea and millet from northern Cameroon: part I. *Food Addit. Contam. Part B Surveill.* **2010**, *3*, 178–184.
119. Soydan, D.K.; Turgut, N.; Yalçın, M.; Turgut, C.; Karakuş, P.B.K. Evaluation of pesticide residues in fruits and vegetables from the Aegean region of Turkey and assessment of risk to consumers. *Environ. Sci. Pollut. Res.* **2021**, *28*, 27511–27519.
120. Srivastava, A.K.; Trivedi, P.; Srivastava, M.K.; Lohani, M.; Srivastava, L.P. Monitoring of pesticide residues in market basket samples of vegetable from Lucknow City, India: QuEChERS method. *Environ. Monit. Assess.* **2011**, *176*, 465–472.
121. Sungur, Ş.; Tunur, Ç. Investigation of pesticide residues in vegetables and fruits grown in various regions of Hatay, Turkey. *Addit. Contam. Part B Surveill.* **2012**, *5*, 265–267.
122. Swarnam, T.P.; Velmurugan, A. Pesticide residues in vegetable samples from the Andaman Islands, India. *Environ. Monit. Assess.* **2013**, *185*, 6119–6127.
123. Szpyrka, E.; Kurdziel, A.; Matyaszek, A.; Podbielska, M.; Rupar, J.; Słowik-Borowiec, M. Evaluation of pesticide residues in fruits and vegetables from the region of south-eastern Poland. *Food Control.* **2015**, *48*, 137–142.
124. Tao, Y.; Jia, C.; Jing, J.; Zhang, J.; Yu, P.; He, M.; Wu, J.; Chen, L.; Zhao, E. Occurrence and dietary risk assessment of 37 pesticides in wheat fields in the suburbs of Beijing, China. *Food Chem.* **2021**, *350*, 129245.
125. Tong, J.; Feng, D.; Wang, X.; Wang, M.; Chen, M.; Chen, Y.; Ma, Y.; Mei, B.; Chen, R.; Gao, M.; Shen, S.; Wang, H.; Zhang, W. Pesticide residue and dietary intake risk of vegetables grown in Shanghai under modern urban agriculture in 2018-2021. *Heliyon.* **2024**, e25505.
126. Toptanci, İ.; Kiralan, M.; Ramadan, M.F. Levels of pesticide residues in fruits and vegetables in the Turkish domestic markets. *Environ. Sci. Pollut. Res. Int.* **2021**, *28*, 39451–39457.
127. Tripathy, V.; Sharma, K.K.; Sharma, K.; Gupta, R.; Yadav, R.; Singh, G.; Aggarwal, A.; Walia, S. Monitoring and dietary risk assessment of pesticide residues in brinjal, capsicum, tomato, and cucurbits grown in Northern and Western regions of India. *J. Food Compos. Anal.* **2022**, *110*, 104543.

128. Walorczyk, S.; Drożdżyński, D.; Kowalska, J.; Remlein-Starosta, D.; Ziółkowski, A.; Przewoźniak, M.; Gnusowski, B. Pesticide residues determination in Polish organic crops in 2007–2010 applying gas chromatography–tandem quadrupole mass spectrometry. *Food Chem.* **2013**, *139*, 482–487.
129. Wang, D.; Yang, G.; Yun, X.; Luo, T.; Guo, H.; Pan, L.; Du, W.; Wang, Y.; Wang, Q.; Wang, P.; Zhang, Q.; Li, Y.; Lin, N. Carbendazim residue in plant-based foods in China: Consecutive surveys from 2011 to 2020. *Environ. Sci. Ecotech.* **2024**, *17*, 100301.
130. Wang, R.; Yang, Y.; Deng, Y.; Hu, D.; Lu, P. Multiresidue analysis and dietary risk assessment of pesticides in eight minor vegetables from Guizhou, China. *Food Chem.* **2022**, *380*, 131863.
131. Wang, S.; Wang, Z.; Zhang, Y.; Wang, J.; Guo, R. Pesticide residues in market foods in Shaanxi Province of China in 2010. *Food Chem.* **2013**, *138*, 2016–2025.
132. Witczak, A.; Pohoryło, A.; Abdel-Gawad, H.; Cybulski, J. Residues of some organophosphorus pesticides on and in fruits and vegetables available in Poland, an assessment based on the European union regulations and health assessment for human populations. *Phosphorus Sulfur Silicon Relat. Elem.* **2018**, *193*, 711–720.
133. Xu, X.; Chen, J.; Li, B.; Tang, L. Carbendazim residues in vegetables in China between 2014 and 2016 and a chronic carbendazim exposure risk assessment. *Food Control.* **2018**, *91*, 20–25.
134. Xu, X.; Li, L.; Huang, X.; Lin, H.; Liu, G.; Xu, D.; Jiang, J. Survey of four groups of cumulative pesticide residues in 12 vegetables in 15 provinces in China. *J. Food Prot.* **2018**, *81*, 377–385.
135. Yi, Y.-J.; Joung, H.-J.; Kum, J.-Y.; Hwang, i-S.; Kim, M.S. Pesticide residues in vegetables and risk assessment for consumers in Korea during 2010–2014. *Food Addit. Contam. Part A Chem. Anal. Control Expo. Risk Assess.* **2020**, *37*, 1300–1313.
136. Yu, R.; Liu, Q.; Liu, J.; Wang, Q.; Wang, Y. Concentrations of organophosphorus pesticides in fresh vegetables and related human health risk assessment in Changchun, Northeast China. *Food Control.* **2016**, *60*, 353–360.
137. Yu, Y.; Hu, S.; Yang, Y.; Zhao, X.; Xue, J.; Zhang, J.; Gao, S.; Yang, A. Successive monitoring surveys of selected banned and restricted pesticide residues in vegetables from the northwest region of China from 2011 to 2013. *BMC Public Health.* **2018**, *18*, 91.
138. Yuan, Y.; Chen, C.; Zheng, C.; Wang, X.; Yang, G.; Wang, Q.; Zhang, Z. Residue of chlorpyrifos and cypermethrin in vegetables and probabilistic exposure assessment for consumers in Zhejiang Province, China. *Food Control.* **2014**, *36*, 63–68.
139. Zhang, Q.; Ma, C.; Duan, Y.; Wu, X.; Lv, D.; Luo, J. Determination and dietary intake risk assessment of 35 pesticide residues in cowpea (*Vigna unguiculata* [L.] Walp) from Hainan province, China. *Sci. Rep.* **2022**, *12*, 5523.
